# Large-scale probabilistic identification of boreal peatlands using Google Earth Engine, open-access satellite data, and machine learning

**DOI:** 10.1371/journal.pone.0218165

**Published:** 2019-06-17

**Authors:** Evan Ross DeLancey, Jahan Kariyeva, Jason T. Bried, Jennifer N. Hird

**Affiliations:** 1 Alberta Biodiversity Monitoring Institute, University of Alberta, Edmonton, Alberta, Canada; 2 Department of Geography, University of Calgary, Calgary, Alberta, Canada; Indiana State University, UNITED STATES

## Abstract

Freely-available satellite data streams and the ability to process these data on cloud-computing platforms such as Google Earth Engine have made frequent, large-scale landcover mapping at high resolution a real possibility. In this paper we apply these technologies, along with machine learning, to the mapping of peatlands–a landcover class that is critical for preserving biodiversity, helping to address climate change impacts, and providing ecosystem services, e.g., carbon storage–in the Boreal Forest Natural Region of Alberta, Canada. We outline a data-driven, scientific framework that: compiles large amounts of Earth observation data sets (radar, optical, and LiDAR); examines the extracted variables for suitability in peatland modelling; optimizes model parameterization; and finally, predicts peatland occurrence across a large boreal area (397, 958 km^2^) of Alberta at 10 m spatial resolution (equalling 3.9 billion pixels across Alberta). The resulting peatland occurrence model shows an accuracy of 87% and a kappa statistic of 0.57 when compared to our validation data set. Differentiating peatlands from mineral wetlands achieved an accuracy of 69% and kappa statistic of 0.37. This data-driven approach is applicable at large geopolitical scales (e.g., provincial, national) for wetland and landcover inventories that support long-term, responsible resource management.

## Introduction

### Peatlands overview and mapping

Wetland ecosystems are of critical importance, not only for the role they play in moderating overland water flow and subsequent flooding [1], and in filtering out freshwater pollutants and sediments, but also as biodiversity hotspots that support a wide range of flora and fauna [2]. Comprehensive mapping and inventory of wetland location, extent, and abundance is essential for planning and management to optimize services and meet the challenges and directives of major conservation initiatives [3]. This is particularly true of boreal peatlands (any wetland that accumulates partially decayed organic matter) which cover large expanses of northern Europe, Canada, and Russia [2] and contribute significantly to global carbon storage and the pace of modern climate change [1, 4]. Carbon sequestered from the atmosphere by photosynthesizing vegetation becomes locked up in accumulated peat when vegetation dies but does not completely decompose [5]. Millennia of peat accumulation has resulted in a global peatland carbon sink that is estimated to exceed that currently stored in global living vegetation [6]. However, peatland carbon cycles and storage can be disrupted, and in some cases transition to a carbon source, by the lowering of water tables resulting from drainage or natural drying, changes in air and soil temperature, or landscape disturbance such as fire or human activities [2, 6–8]. The effects of human disturbance and climate change on peatland function and carbon fluxes are therefore of great importance and interest. An important element in understanding these effects and their implications for future sustainability of peatland environments is first having accurate, up-to-date knowledge of where they are on the landscape.

Peatlands can be broadly divided into two classes: 1) acidic, nutrient-poor bogs, which are dominated by peat moss and are closed to surface water or groundwater flow (i.e., their sole source of water is precipitation); and 2) nutrient-rich, minerotrophic fens that are covered by graminoid vegetation and are open to surface water or groundwater flow [9]. Beyond these two basic types, however, peatland classifications are complex and geographically variable, making it difficult to define mapping units [10]. Their large geographic extent, natural heterogeneity, and cultural and socio-economic value makes accurate identification and mapping of peatlands both critical and challenging.

Canada supports one of the world’s largest extents (>1 million km^2^) of peatlands and peat resources [9], which comprises approximately 12% of its total land area, and 27% of global peatlands [11]. Peatland mapping in Canada has traditionally been accomplished through two approaches: 1) photo-interpreted or modeled, vector-based inventories [12, 13]; and 2) coarse scale, remotely-sensed landcover classification [14, 15]. Photo interpretation accuracy is often limited by the quality and temporal availability of the source imagery (leaf-off color infrared photography is best for wetland mapping [10]). Optical, remotely-sensed landcover classification (e.g., using MODIS or Landsat data) often ignores the underlying hydrology that drives wetland formation, structure, and function [16]. Recently, hybrid approaches to wetland mapping that include optical, radar, and topographical inputs have emerged with more promising results; likely due to the use of derivatives from digital elevation models (DEM) which provide additional information on local hydrology patterns [17–21]. Tracking dynamic wetland hydroperiods in near-real time is also now possible with high temporal resolution SAR data [22, 23], but hydrodynamics are typically underrepresented or absent in large-scale wetland inventories.

### Advances in remote sensing and data science

Recent advances and developments in open-access satellite data streams, cloud computing, and data science have made large-scale, high-resolution landcover classifications more feasible for a broad set of user groups, organizations, and researchers [21, 22, 24, 25]. The processing and analysis of open-source satellite data has been revolutionized with cloud-based platforms such as Google Earth Engine (GEE) [25]. GEE stores multi-petabyte satellite data streams and allows for the easy access and processing of this data (through parallel computation service) using a simple internet-accessible Application Programming Interface [25]. At the same time, advances in open-source data science algorithms and packages in R and Python (e.g., TensorFlow, Keras, dplyr, ggplot2, Altair, RStoolbox, dismo) have enabled detailed analysis and modelling of vast amounts of open-source satellite data [26–30]. This combination of easily-accessible satellite data, and powerful data analysis, visualization, geocomputation, and modelling tools/packages, has dramatically increased our ability to produce up-to-date landcover classifications across large regions.

Satellite earth observation provides synoptic and repeating views of the Earth’s surface and is therefore well-recognized as a key data source for the large-scale mapping and monitoring of a wide-range of ecosystem functions and services [16, 31, 32]. Optical sensors offer information on vegetation cover and community type, which can be used to identify and differentiate wetlands and vegetation zones and have shown potential for mapping peatlands at cold-temperate and subarctic latitudes [33–35]. However, optical sensors are limited to daytime image acquisition and by their inability to penetrate through cloud and atmospheric haze or dense vegetation canopies [36]. Unlike optical sensors, active radar sensors (Synthetic Aperture Radar; SAR) are not limited by atmospheric conditions, can detect sub-canopy soil and vegetation structural features, and are not reliant on external sources of radiation (i.e., sunlight). They have proven to be a useful alternative or supplemental source to optical images for peatland mapping [37–39]. These data, however, are often subject to the effects of surface moisture content and roughness, and instrument viewing direction and incidence angle [36]. It is therefore the combination of optical and radar satellite data which offers the greatest potential for supporting peatland mapping and monitoring, as described by [40].

### Objectives

Here we build on the work of [21] where wetland extent was mapped in a 13,700 km^2^ region of the Boreal Forest Natural Region of Alberta (BNR) with Sentinel-1 (SAR), Sentinel-2 (optical), and topographic data with promising results in terms of accuracy (85%), spatial resolution (10 m), and large-area scalability. We expand on [21] by providing more information on wetland type, and by establishing a data processing framework wherein large amounts of Earth observation data from different sources can be used to classify landcover at a high spatial resolution (e.g., 10 m), over larger areas (397, 958 km^2^) with relatively high frequency. Taking general wetland location across the BNR of Alberta from [21], we further separate peatland from non-peatland including uplands and mineral wetlands. Mineral wetlands are characterized by soils with < 17% organic carbon and peat < 40 cm in thickness [41]. Current landcover inventories in Alberta are either spatially inconsistent across the province (Derived Ecosite Phase [42]; Alberta Vegetation Inventory [43]) or are provided as lower spatial resolution products [12, 15]. We hope that our framework contributes not only to improved wetland mapping in Alberta (i.e., large-scale, high-resolution, spatially-consistent) and therefore, supports better understandings of the current state of Alberta’s peatlands, but also to building a state-of-the-science, data-driven mapping framework for any landcover mapping project.

## Methods

### Study area

**Fig 1:**
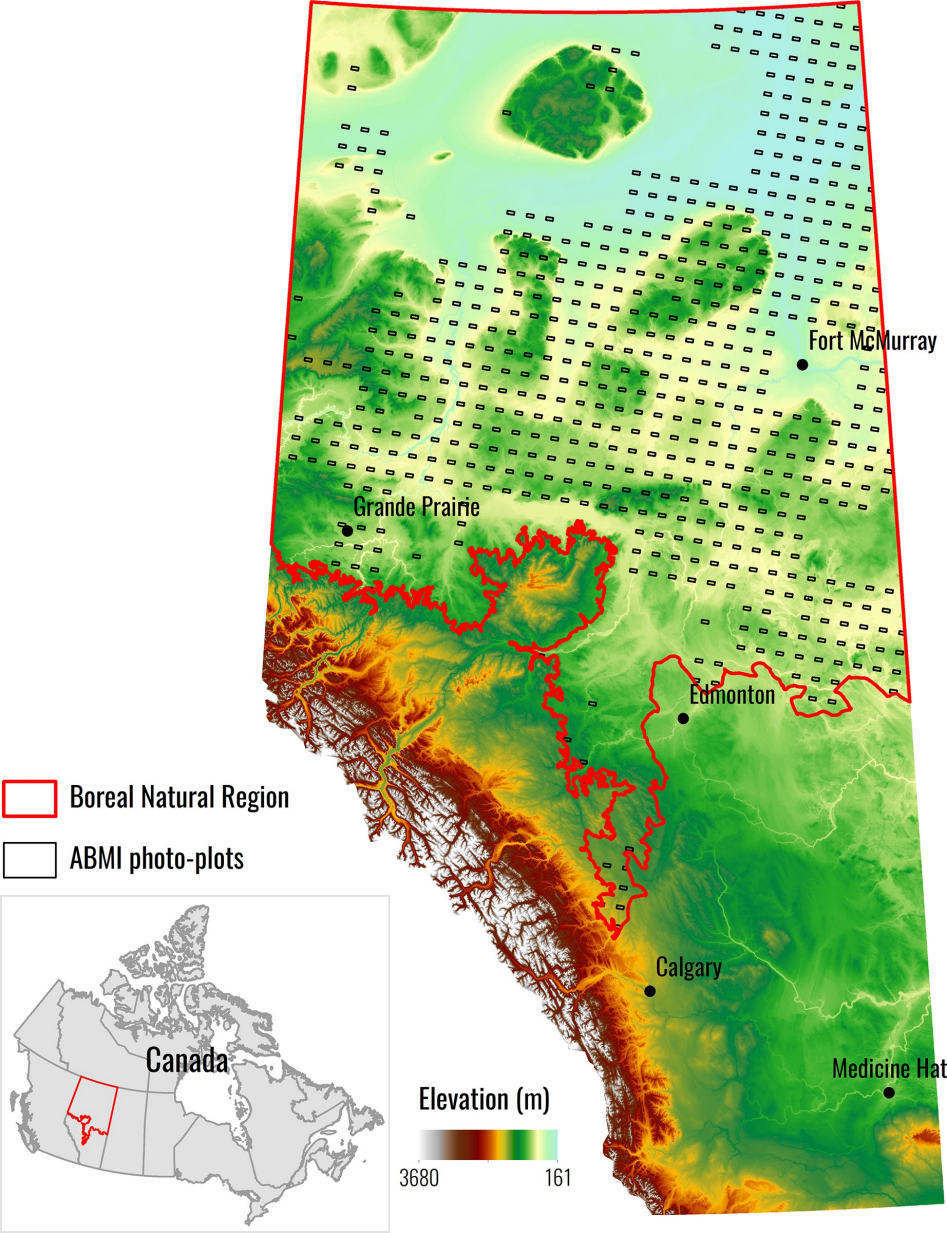
Location of the Boreal Forest Natural Region and the ABMI photo-plots used for training and validation, underlain by elevation. The ABMI has given permission to publish this image under a CC BY 4.0 license.

Our study area includes the BNR of Alberta, Canada along with small parts of the Canadian Shield, Parkland, and Foothills Natural Regions to form a continuous area (Fig 1). This study area comprises approximately 60% (397, 958 km^2^) of the total area of Alberta. Elevations range from 150 m above sea level in the northeast to 1,100 m near the Alberta-British Columbia border [44].

The BNR has short summers and long, cold winters [44]. Vegetation is primarily in the form of vast deciduous, mixedwood, and coniferous forests interspersed with extensive wetlands [44]. Agriculture is limited to the southeast region of the study area (northeast of Edmonton, a large urban center) and areas around Grand Prairie (western portion of study area) [45]. Other anthropogenic features come in the form of forestry activities and extensive oil and gas development in the regions around Fort McMurray [45].

The Alberta Wetland Classification System recognizes five main wetland classes across the province: bog, fen, marsh, swamp, and shallow open water [13, 41]. Bogs, fens, and occasionally swamps (>25% tree cover) are classified as peatlands in Alberta [46]. Peatlands usually contain extensive cover of bryophytes (especially *Sphagnum* spp) with limited areas of open water [46]. The BNR is dominated by fens and bogs, which typically form in cool, flat, low-lying areas with poorly drained soils and peat accumulations of 30–40 cm or more [5, 47]. The fens and bogs of this region are classified as wooded coniferous, shrubby, or graminoid with bogs being relatively acidic and fens ranging from poor acidic to extreme-rich alkaline [41, 48]. Our analysis focuses on mapping the occurrence of all types of fens and bogs in the BNR.

### Data

Sentinel-1, -2 (S1 and S2 [49]); LiDAR-derived digital elevation model (DEM [50]); and Shuttle Radar Topography Mission (SRTM) DEM [51] data were used to generate a peatland probability model in the BNR. All Sentinel and SRTM data were acquired, processed, and downloaded through GEE (See Supporting Information for source JavaScript code) [25]. GEE stores S1 (SAR imagery) ground range-detected scenes which have been pre-processed with the Sentinel-1 Toolbox (Sentinel Application Platform–Sentinel-1 Toolbox). These pre-processing steps include thermal noise removal, radiometric calibration, and terrain correction. Sentinel-1 data contains a VV band–vertical polarization sending and vertical polarization receiving–and a VH band–vertical polarization sending and horizontal polarization receiving. Dual polarization (VV and VH polarizations) S1 images during leaf-on season (May 15 –August 31) were further processed in the GEE environment by performing an incidence angle correction [52] and smoothing with a 3x3 Sigma Lee filter [53] (credit to Guido Lemoine for GEE code). Once all S1 images were processed, a normalized difference of polarization (NDPOL) was calculated (see Table 1) and added to the available bands. To generate a single composite image for the S1 variables, the per-pixel mean of the VH and NDPOL bands were calculated. A total of 478 S1 images were used in the calculation of the VH and NDPOL variables.

**Table 1:** Candidate input variables for the peatland probability model.

Variable	Data source	Equation	Description
ARI	Sentinel-2	$\left(\frac{Band\;8}{Band\;2}\right)-\left(\frac{Band\;8}{Band\;3}\right)$	Anthocyanin Reflectance Index. An index sensitive to anthocyanin pigments in plant foliage which is often associated with plant stress or senescence [56].
NDVI	Sentinel-2	$\frac{\left(Band\;8-Band\;4\right)}{\left(Band\;8+Band\;4\right)}$	Normalized Difference Vegetation Index. Index for estimating photosynthetic activity, and leaf area [57].
NDWI	Sentinel-2	$\frac{\left(Band\;3-Band\;8\right)}{\left(Band\;3+Band\;8\right)}$	Normalized difference Water Index from [58].
NDPOL	Sentinel-1	$\frac{\left(VH-VV\right)}{\left(VH+VV\right)}$	Normalized Difference of Polarization and index used to measure SAR double bounce off of flooded vegetation.
PC1	Sentinel-2	-	The first principal component of variation of Bands 2, 3, 4, and 8 of Sentinel-2 data.
PC2	Sentinel-2	-	The second principal component of variation of Bands 2, 3, 4, and 8 of Sentinel-2 data.
PSRI	Sentinel-2	$\frac{\left(Band\;4-Band\;2\right)}{\left(Band\;5\right)}$	Plant Senescence Reflectance Index. A ratio used to estimate the ratio of bulk carotenoids to chlorophyll [59].
REIP	Sentinel-2	$702+40\left(\frac{\left(\frac{Band\;4+Band\;7}{2}\right)-Band\;5}{\left(Band\;6-Band\;5\right)}\right)$	Red Edge Inflection Point. An approximation on a hyperspectral index for estimating the position (in nm) of the NIR/red inflection point in vegetation spectra [60].
TPI	LiDAR, SRTM DEMs	-	Topographic Position Index (TPI) generated in SAGA [55]. An index describing the relative position of a pixel within a valley, ridge top continuum calculated in a given window size. TPI was calculated with a 500m moving window for this purpose [61].
TPI_SRTM	SRTM DEM	-	Same index as TPI (above) but only calculated with SRTM data.
TWI	LiDAR, SRTM DEMs	-	Saga Wetness Index. A SAGA [55] version of the Topographic Wetness Index. Potential wetness of the ground based on topography [62].
TWI_SRTM	SRTM DEM		Same index as TWI (above) but only calculated with SRTM data.
VH	Sentinel-1	-	Vertical polarization sending horizontal polarization receiving SAR backscatter in decibels.

Sentinel-2 (optical imagery) top-of-atmosphere data was also accessed through GEE. Clouds, shadows, snow, and ice were flagged using the provided QA60 band–a quality control band provided by the European Space Agency used to identify cloud/cloud shadow pixels–and removed, while further cloud masking was performed using a threshold with S2 band 1 (band 1>1500). A total of 3,148 S2 images, intersecting with the BNR during the 2016–2017 leaf-on season (May 15 –August 31), were used to extract 10 m spectral bands (B2, B3, B4, and B8) and generate vegetation indices. Bands 2,3,4, and 8 were put into a Principal Component Analysis [29] and transformed into the first two principal components of variation to reduce the number of modelling variables. The first principal component contained 73% of the variance, while the second component contained 24% of the total variance. Generally, this Principal Component method provides a good method for reducing data inputs and correlation between inputs. The final S2 input layers were generated using a variable-by-variable, pixel-based median composting algorithm where the median time series value for each pixel was selected as the most representative pixel for that time period.

**Fig 2:**
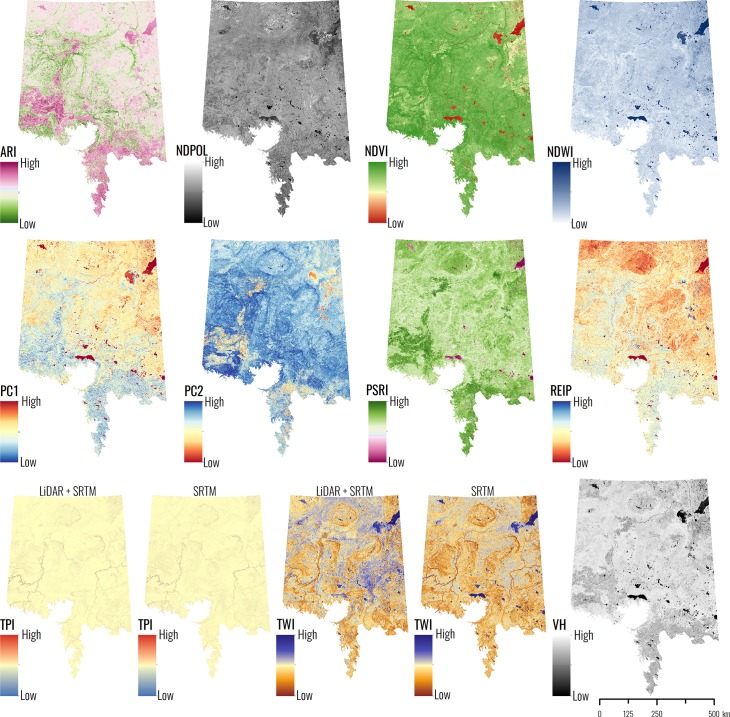
All 13 candidate input variables across the entire study area. Two different versions of TPI and TWI are shown since one version was calculated with LiDAR + SRTM topographic data and one was calculated with just SRTM topographic data. The SRTM derived version is noted by the “_SRTM” in the variable name. The ABMI has given permission to publish this image under a CC BY 4.0 license.

The topographic data used for modelling originated from three sources: 1) a 1 m bare earth LiDAR-derived DEM covering the forested regions of Alberta [50]; 2) a 15 m bare earth LiDAR-derived DEM covering the prairie regions of Alberta [54]; and 3) a 30 m SRTM DEM used to fill in gaps where the previous two sources do not provide coverage [51]. The 1 m LiDAR-based DEM data set was mean aggregated to 10 m to match the S1 and S2 spatial resolutions, whereas the 15 m LiDAR-based DEM was resampled to 10 m using a cubic convolution method. The 30 m SRTM data was converted into a floating-point raster, then resampled to 10 m using cubic convolution, and subsequently smoothed using a 7 pixel x 7 pixel spatial mean filter. This smoothing was done to better match the indices produced by the LiDAR-based data. Two topographic indices as seen in [21] (TWI and TPI, Table 1) were calculated separately for each topographic data set and then merged when complete. All topographic indices were calculated in SAGA version 5.0.0 [55]. All model input variables are presented in Fig 2, while equations and description are provided in Table 1. For all the websites and databases in which we collected data from, the proper terms and conditions were followed.

Training and validation data were independently extracted from the Alberta Biodiversity Monitoring Institute (ABMI) 3x7 km Landcover Photo-plots (hereafter ABMI plots [63]) (see Fig 1 for spatial distribution). These photo-plots are derived from high resolution 3D image interpretation and provide a detailed attribution of landcover information that includes nine moisture classes, 22 tree species classes, and 28 modified wetland classes [63]. The ABMI plots have undergone ground-truthing with extensive field work. The photo interpretations are usually highly accurate (high 90%) when compared to the field data. These data sets cover approximately five percent of the total area of Alberta, and are typically very accurate, with less than 1% of features possessing errors based on independent interpretation audits [63].

### Data exploration, variable selection and model optimization

To explore 13 candidate input variables (Table 1 and Fig 2) for use in our peatland probability model, we generated 200,000 random points within the ABMI plots. For each point, the 13 input variable values were extracted, producing a data frame with 14 columns (one for peatland vs. mineral wetland classification) and 200,000 entries. To visualize the predictive power of each variable, a violin plot (ggplot2 [64]) was generated to compare each variable in peatland and mineral wetland classes. To assess variable importance inside a model, a single Boosted Regression Tree model (BRT [65]) was run using 50,000 random points and the relative variable importance in the model was examined. To minimize multicollinearity, we sequentially worked through the variable importance list and removed those variables that had a high correlation (Pearson’s r > 0.7) with any of the high importance variables.

The next step was to select optimal parameters for our BRT model, which is a machine learning algorithm that employs decision trees and boosting [65] (results of this can be seen in Supporting information). There are two parameters that can be altered within this algorithm to fit one’s model: tree complexity (the number of nodes in a tree) and learning rate (the contribution of each tree to the final model). We iteratively altered the learning rate, tree complexity, and number of modelling variables, selecting optimal parameter values based on the Area Under the Receiver Operating Characteristic Curve (AUROC) statistic, explained deviance, and percent accuracy when compared with an independent training sample derived from the ABMI photo-plots. Additionally, we estimated the optimal number of training samples by varying the number of training samples from 407 to 91,347 in the BRT model and checking the accuracy of the resulting classification in comparison to an independent training source. Forty iterations of each test were conducted using different sets of training samples (derived from the ABMI plots), as was done in our modeling methods described below.

### Wetland classification–machine learning algorithm and spatial prediction

To model peatland probability within our study area, a BRT machine learning algorithm was implemented using the dismo package available in the R Statistical Software (See Supporting Information for R source code) [15, 21, 30, 65, 66]. To build our model, 6,497 random points (see Supporting Information for justification) within the ABMI plots were split equally between peatland and mineral wetland classes and placed at a minimum distance of 375 m from one another in known wetland areas as indicted by the ABMI Wetland probability data set–a landcover data set describing the location and extent of wetlands in Alberta [67]. Training points were not placed in any locations within known human footprint features, or areas with open water based on spatial delineations from [45, 68]. The peatland/mineral wetland training data itself was extracted from the ABMI plots. For the purposes of training our model, fen and bog classes from the ABMI plot data were reclassified as peatlands while marsh, swamp, and shallow open water classes were reclassified as mineral wetlands. The training data set comprising these 6,497 points was then passed into a BRT modelling function where tree complexity was set to 8 and learning rate to 0.005 (see Supporting information). Model outputs included: responses for the input variables, variable importance, an AUROC value, and explained deviance. The model was then applied to the study area to predict peatland probability across the BNR given the input variables. This process was repeated for 40 iterations so as to reduce statistical overfitting and spatial auto-correlation [69], and generated 40 peatland probability grids. The per-pixel mean value of these 40 probability surfaces provided our final peatland probability surface. Uncertainty among our 40 models was assessed by calculating the standard deviation in peatland probability for each pixel across the 40 iterations. Peatlands were then classified as any value above a probability threshold of 0.5 resulting in a binary peatland (1)/mineral wetland (0) raster. A 0.5 threshold was chosen as this was found to provide the highest accuracy and highest kappa value for all threshold values.

Once peatland probability was predicted across the BNR, areas with surface water [68], human footprint [45], or upland [67] were given a peatland probability value of 0. The final probability raster was converted into a binary peatland/mineral wetland raster and smoothed using a 5x5 majority filter to smooth boundaries between classes and remove the “salt and pepper” appearance. Finally, a traditional four class (i.e., open water (lakes, ponds, rivers), upland, peatland, mineral wetland) landcover data set was created by combining the ABMI surface water and uplands data sets [67, 68] with the peatland/mineral wetland data produced using the methods described above.

### Cross-validation accuracy assessment

An independent cross-validation accuracy assessment of the binary peatland/mineral wetland raster was completed by generating 200,000 points in all of the ABMI plot areas within the BNR. While the training and validation data are from the same source (i.e., ABMI plots), each was generated independently and points from the training data are not found in the validation data set. Each 10 m pixel was classified as peatland or non-peatland (e.g., water, upland, mineral wetland). Values from the ABMI plot validation data and the modeled peatland data were then extracted for each point. With these data, an area adjusted accuracy assessment and confusions matrix was calculated following the methods from [70].

An additional accuracy assessment was done within wetland areas themselves to assess the capability of our model to differentiate peatlands from mineral wetlands. Again, 200,000 points were generated inside the BNR wetland areas, and an area adjusted accuracy assessment and confusions matrix was calculated following the methods from [70].

## Results

### Data exploration and variable selection

**Fig 3:**
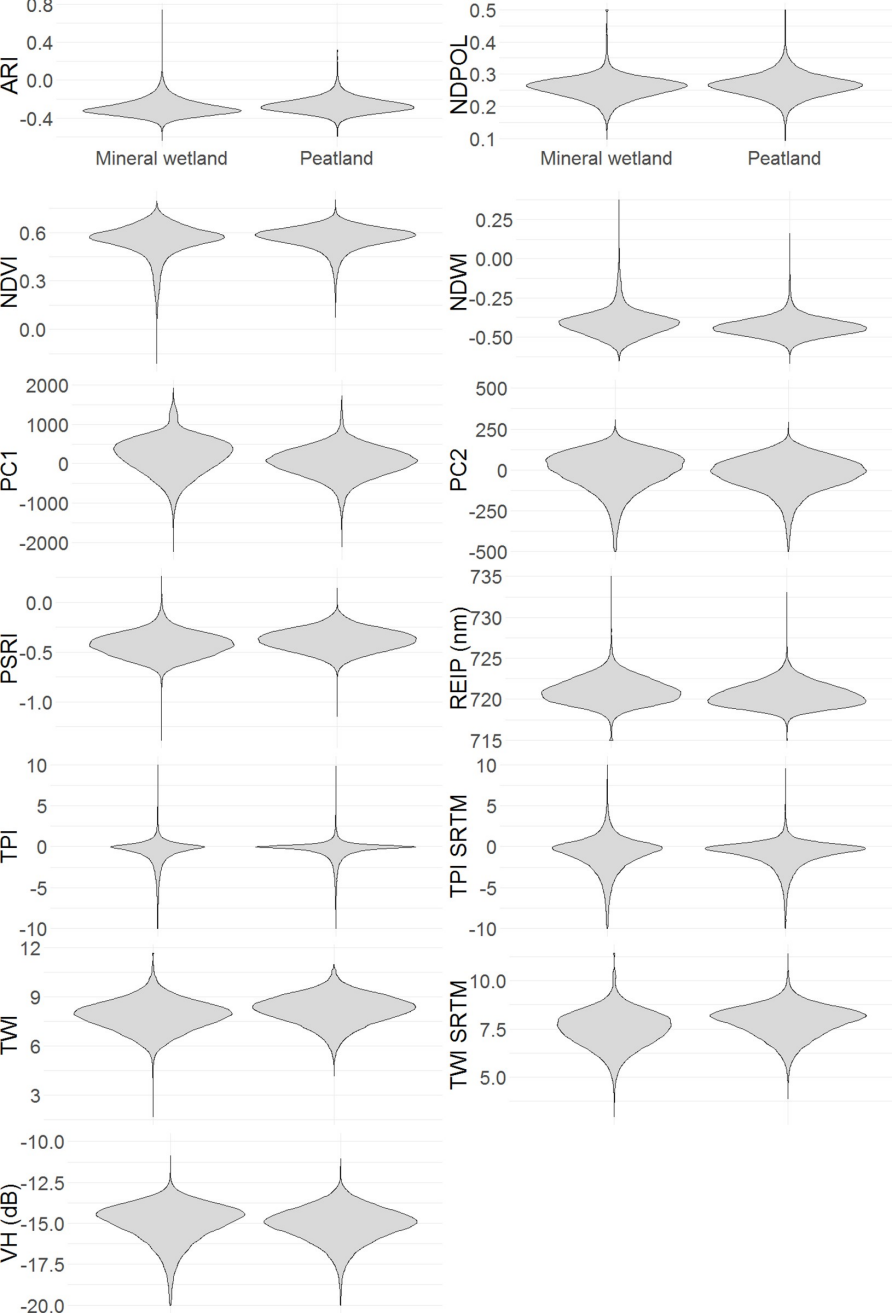
The distribution of values by peatland and mineral wetland classes for the 13 possible input variables in the peatland occurrence model. The ABMI has given permission to publish this image under a CC BY 4.0 license.

Fig 3 shows distributions for each candidate model input variables in the peatland and mineral wetland classes. The majority of plots show small differences between the classes. The largest difference can be seen in the PC1, TWI, and VH variables (see Table 1 for definitions), which suggests these variables have greater potential to discriminate between the two classes. The TPI panel shows that peatlands have a much larger proportion of values around zero than mineral wetlands. REIP and TWI have the strongest correlation to peatland occurrence (*r* = 0.21 and 0.20 respectively;Table 2). Many of the Sentinel-2 variables were strongly correlated with one another (*r* > 0.60) while the NDPOL, TPI, and TWI variables showed very little correlation with other variables and therefore are seen as the most unique variables (Table 2). We retained REIP, PC1, TWI, TPI, NDPOL, ARI, VH, and PC2 for the peatland model based on their relative importance (Table 3), and to mitigate collinearities (Table 2; i.e. *r* < 0.7 among any variable pairs). It should be noted that TWI and TPI were selected over TWI_SRTM and TPI_SRTM since TWI and TPI have a higher native resolution (1 m resampled to 10 m versus 30 m resampled to 10 m). Ultimately, only REIP, PC1, TWI, NDPOL, and ARI were retained for modelling since the addition of subsequent variables (VH, PC2) did not increase the models predictive power (Supporting information).

**Table 2:** Cross correlation (Pearson coefficient) between 13 possible input variables and a binary peatland/mineral wetland grid. PL represents the peatland (1) mineral wetland (0) values. The SRTM version of TWI and TPI were removed since to avoid redundancy with the LiDAR + SRTM derived versions.

	ARI	NDPOL	NDVI	NDWI	PC1	PC2	PSRI	REIP	TPI	TWI	VH	PL
ARI	1	-0.03	0.02	-0.23	-0.57	-0.53	0.69	-0.17	0.00	0.06	-0.40	0.14
NDPOL	-0.03	1	0.06	-0.04	0.02	0.15	-0.01	-0.25	0.08	0.03	0.03	0.08
NDVI	0.02	0.06	1	-0.88	-0.61	0.51	-0.08	0.04	0.08	0.03	0.45	0.10
NDWI	-0.23	-0.04	-0.88	1	0.82	-0.21	-0.29	-0.06	-0.06	-0.07	-0.23	-0.17
PC1	-0.57	0.02	-0.61	0.82	1	0.21	-0.61	-0.09	-0.02	-0.12	0.08	-0.18
PC2	-0.53	0.15	0.51	-0.21	0.21	1	-0.73	0.04	0.08	-0.02	0.58	-0.06
PSRI	0.69	-0.01	-0.08	-0.29	-0.61	-0.73	1	-0.21	-0.02	0.07	-0.51	0.21
REIP	-0.17	-0.25	0.04	-0.06	-0.09	0.04	-0.21	1	-0.11	0.08	0.14	-0.17
TPI	0.00	0.08	0.08	-0.06	-0.02	0.08	-0.02	-0.11	1	0.24	0.07	0.09
TWI	0.06	0.03	0.03	-0.07	-0.12	-0.02	0.07	0.08	0.24	1	-0.06	0.20
VH	-0.40	0.03	0.45	-0.23	0.08	0.58	-0.51	0.14	0.07	-0.06	1	-0.04
PL	0.14	0.08	0.10	-0.17	-0.18	-0.06	0.21	-0.17	0.09	0.20	-0.04	1

**Table 3:** Relative importance of 13 candidate input variables in the BRT peatland occurrence model.

Input variable	Relative importance
TWI_SRTM	11.43
REIP	11.32
PC1	8.98
TWI	8.40
TPI_SRTM	6.99
TPI	6.63
NDPOL	5.73
ARI	5.49
NDWI	5.39
PSRI	5.12
VH	4.11
NDVI	3.88
PC2	1.45

### Peatland classification–machine learning algorithm and spatial prediction

**Fig 4:**
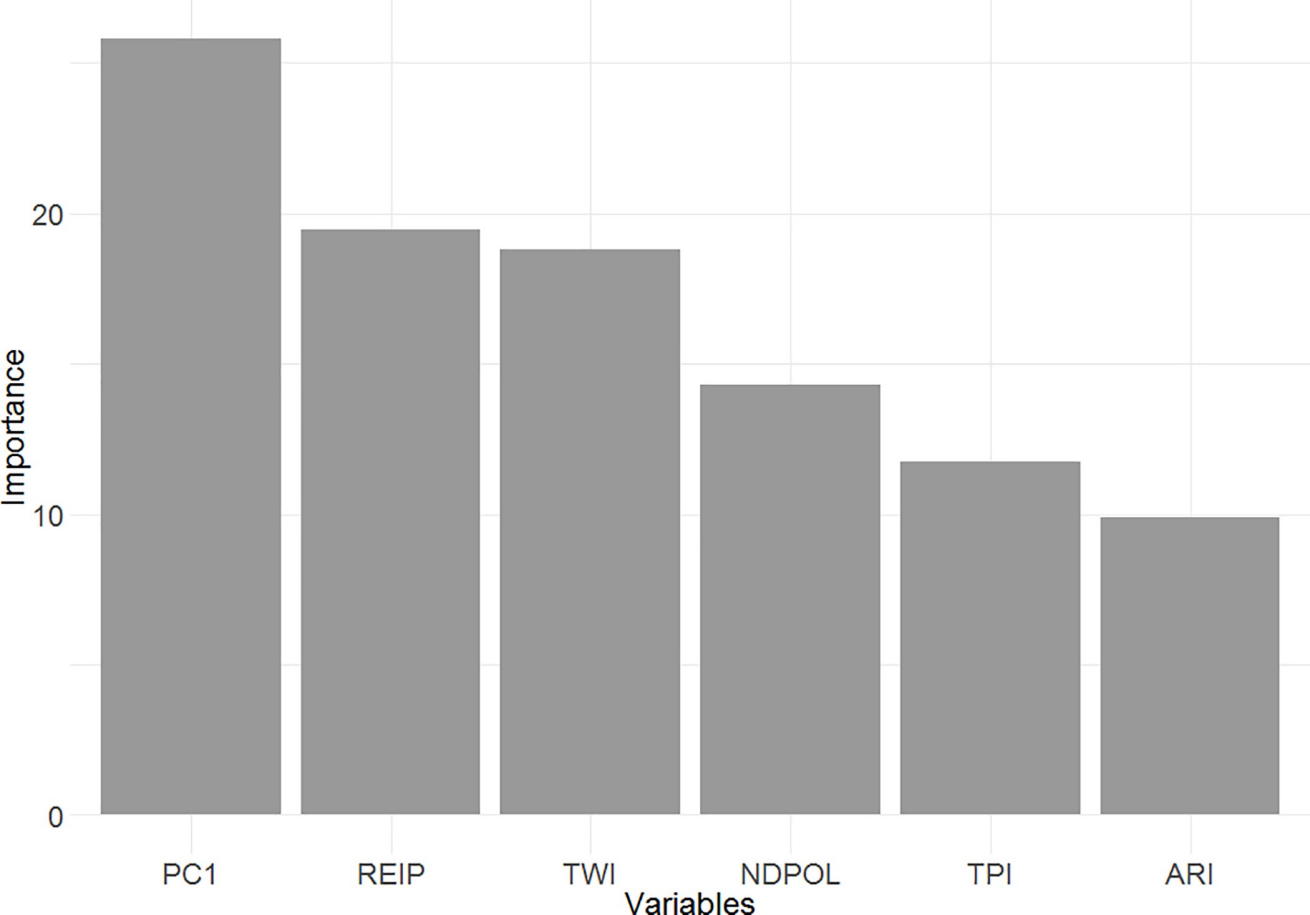
The relative importance of the input variables in the peatland probability model. The ABMI has given permission to publish this image under a CC BY 4.0 license.

**Fig 5:**
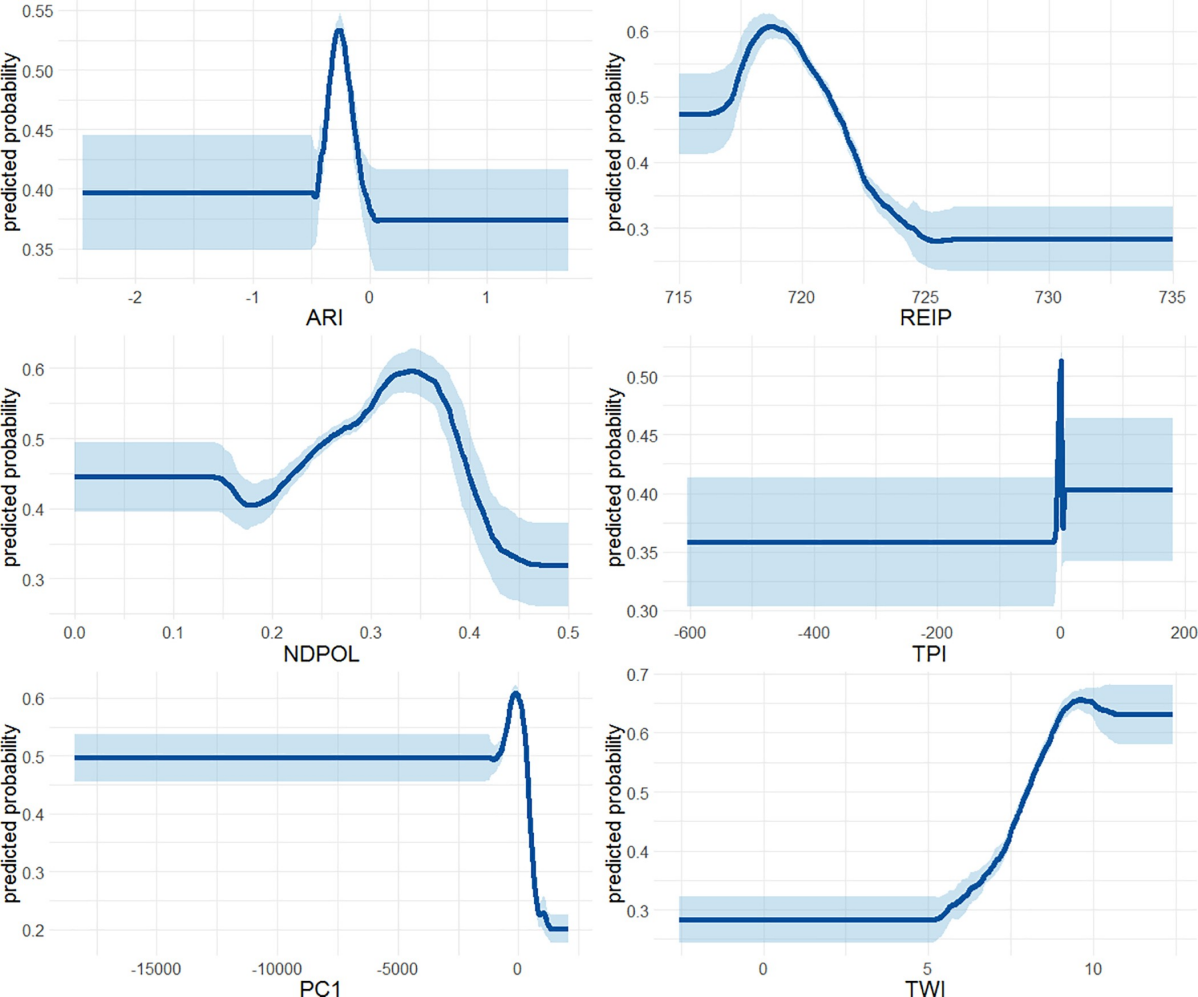
Mean partial dependence response curves for the six input variables in the peatland probability model. The solid line represents the mean response over 40 iterations while the light blue represents the standard deviation of the 40 iterations. The ABMI has given permission to publish this image under a CC BY 4.0 license.

The results of the BRT model show that PC1, REIP, and TWI were relatively important for predicting peatland occurrence (Fig 4). NDPOL, TPI, and ARI were less important in the model but still provided some value (Fig 4). These results are different than that of Table 3 since only six modelling variables were involved in the final BRT model. Overall, the response curves of the variables followed the expected trends (Fig 5). In summary, peatlands had: higher ARI values (higher plant stress), lower NDPOL values (less double bounce backscatter), lower PC1 values (likely higher brightness), lower REIP values (lower photosynthetic activity), spike in probability at TPI = 0 (most likely to occur in very flat regions), and >60% probability of occurrence at TWI > 9 (more likely to occur in topographically wetter areas). The overall AUROC of the model was 0.74 and the explained deviance was 0.21.

**Fig 6:**
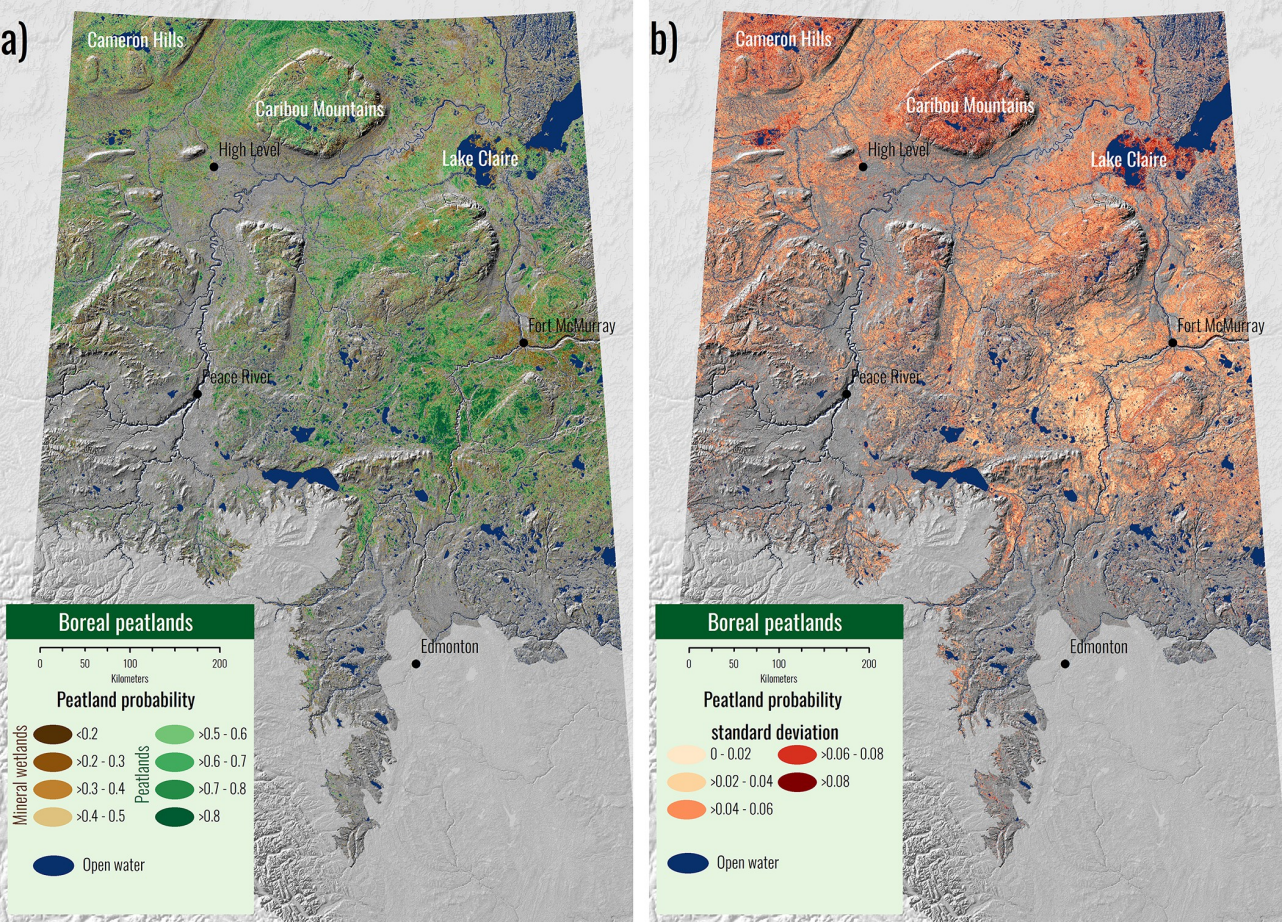
a) Peatland probability model applied across the study area. Greens show peatlands and browns show mineral wetlands. Deeper shades represent a higher probability of either class. Upland areas are not shown in the map and background is a DEM derived hill shade. b) Standard deviation in peatland probability across 40 models. Darker reds represent a higher standard deviation and thus more uncertainty in the classification. Beige represents low standard deviation and higher certainty in the classification. The ABMI has given permission to publish this image under a CC BY 4.0 license.

The model applied across the entire study area predicted very high peatland probability (>0.8) southwest of Fort McMurray (Fig 6A). A continuous region of mineral wetlands can be seen around Fort McMurray and it appears to align with a 2016 wildfire boundary suggesting that the fire strongly affected spectral signature patterns observed in the REIP and ARI variables. Large extents of mixed wetland habitat can be seen in the north-central plateaus of the Caribou Mountains and the Cameron Hills region. The large peatland area southwest of Fort McMurray shows very low variation among 40 models indicating higher model certainty (Fig 6B). In contrast, the regions around Lake Claire, Caribou Mountains and Cameron Hills all show high deviation between models (> 0.10 standard deviation in probability in some cases).

**Fig 7:**
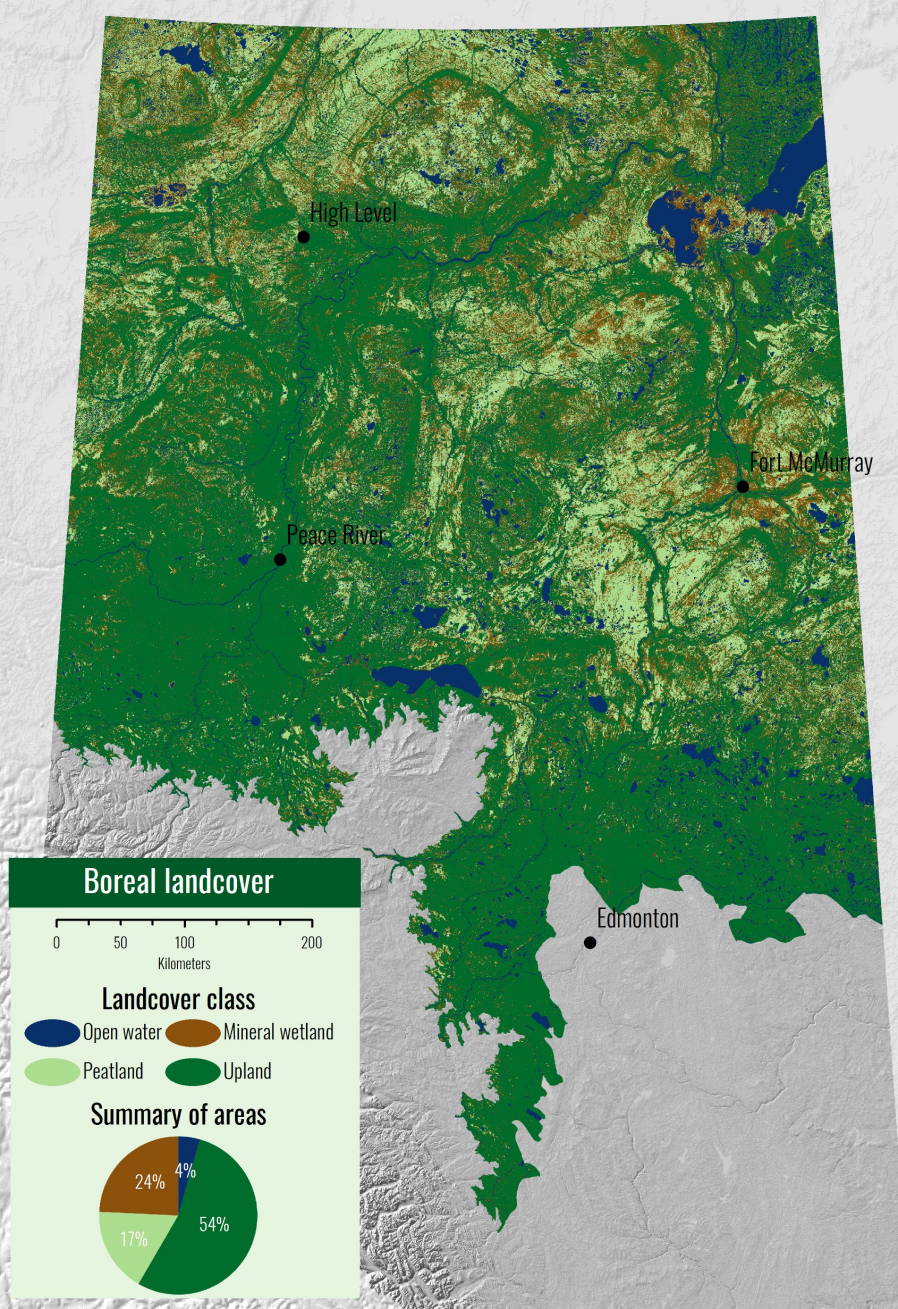
The BNR divided into four landcover classes: open water, mineral wetland, peatland, and upland. The water and upland classes are extracted from the ABMI open water and upland classifications. The peatland and mineral wetland classes are the result of the peatland probability classification described in this study. The ABMI has given permission to publish this image under a CC BY 4.0 license.

Within the individual peatland sub-classes, the resulting map was the best at identifying open and treed bogs (71% and 80% accurate respectively) and the least certain class was open fens and treed fens (57% and 59% respectively). Dividing the study region into four major landcover classes, we see that most of the study area is predicted to be uplands, followed by mineral wetland (marsh, swamp, shallow open water), peatland (fen, bog), and open water (Fig 7).

Using the 0.5 probability threshold, our peatland probability model yields 87% accuracy (0.57 kappa statistic) when classifying peatlands and non-peatlands (Table 4). This essentially tells us that we are easily able to distinguish non-peatland areas. Model performance reduces to 69% accuracy (0.37 kappa statistic) when distinguishing peatlands from just mineral wetlands (Table 5). This demonstrates that peatlands are hard to distinguish from other wetland types. These numbers differ since the first accuracy samples across the whole landscape (uplands and open water are easy to distinguish) while the second accuracy only samples within wetlands. As expected, non-peatland user classification accuracy is much higher than that for peatland (90% vs. 70%, respectively; Table 4). The user accuracy for peatlands and mineral wetlands appeared to be very similar (69% and 68%, respectively; Table 5).

**Table 4:** Area adjusted confusion matrix for the peatland/non-peatland cross validation accuracy assessment.

Class	Non-peatland	Peatland	Total	User’s	Producer’s	Overall
Non-Peatland	0.743	0.080	0.823	0.903 ± 0.001	0.932 ± 0.001	0.866 ± 0.002
Peatland	0.054	0.123	0.177	0.695 ± 0.004	0.607 ± 0.004	
Total	0.797	0.203	1			

**Table 5:** Area adjusted confusion matrix for the cross validation accuracy assessment with only samples inside wetland areas.

Class	Mineral wetland	Peatland	Total	User’s	Producer’s	Overall
Mineral wetland	0.383	0.179	0.562	0.680 ± 0.003	0.739 ± 0.002	0.685 ± 0.002
Peatland	0.135	0.303	0.438	0.692 ± 0.003	0.628 ± 0.002	
Total	0.518	0.482	1			

## Discussion

In this study we achieved a spatially-consistent, large-scale (397, 958 km^2^), high resolution (10 m) probabilistic classification of peatland occurrence, using the Boreal Forest Natural Region of Alberta, Canada as a case example. The method was highly successful at differentiating peatlands from other habitats (87%), and moderately successful at differentiating peatlands from mineral wetlands (69%). We note, however, that peatlands generally occur as a mosaic of wooded, scrub-shrub, and graminoid communities and the method does not distinguish among these or delineate the boundaries. Nevertheless, reliably estimating the amount and configuration of peatlands over large spatial extents is a critical first step to any conservation planning and resource management. The framework presented here offers a new, more pragmatic approach to mapping peatlands across northern boreal regions than what currently exists, and could play a role in future understandings of peatland distributions and long-term monitoring.

With reference to Figs 3, 4 and 5 we can begin to understand how peatlands can be detected (i.e., using their remote sensing signature) in this part of the world. Most importantly, the majority of peatland sub-classes (treed bog, open gramminoid fen, shrub fen, etc.) occur in the most topographically wet areas. While other wetland classes do occur in very topographically wet areas, peatlands typically occur in wet areas that are flat rather than a localized depression–topographic position near or slightly below 0. Peatlands therefore may have a defining “topographic signature”. In addition to the topographic signature, peatlands can be identified from other wetlands due to the lack of SAR double bounce from water to vegetation (NDPOL). This is due to the fact that C-band radar will typically not penetrate deep enough into the peat to bounce off the water table [71, 72]. Peatlands may also be distinguished as they seem to be less photosynthetically active than neighbouring landcover types as they demonstrate higher visible wavelength brightness (PC1; i.e., absorb less sunlight) and lower vegetation productivity (REIP).

**Fig 8:**
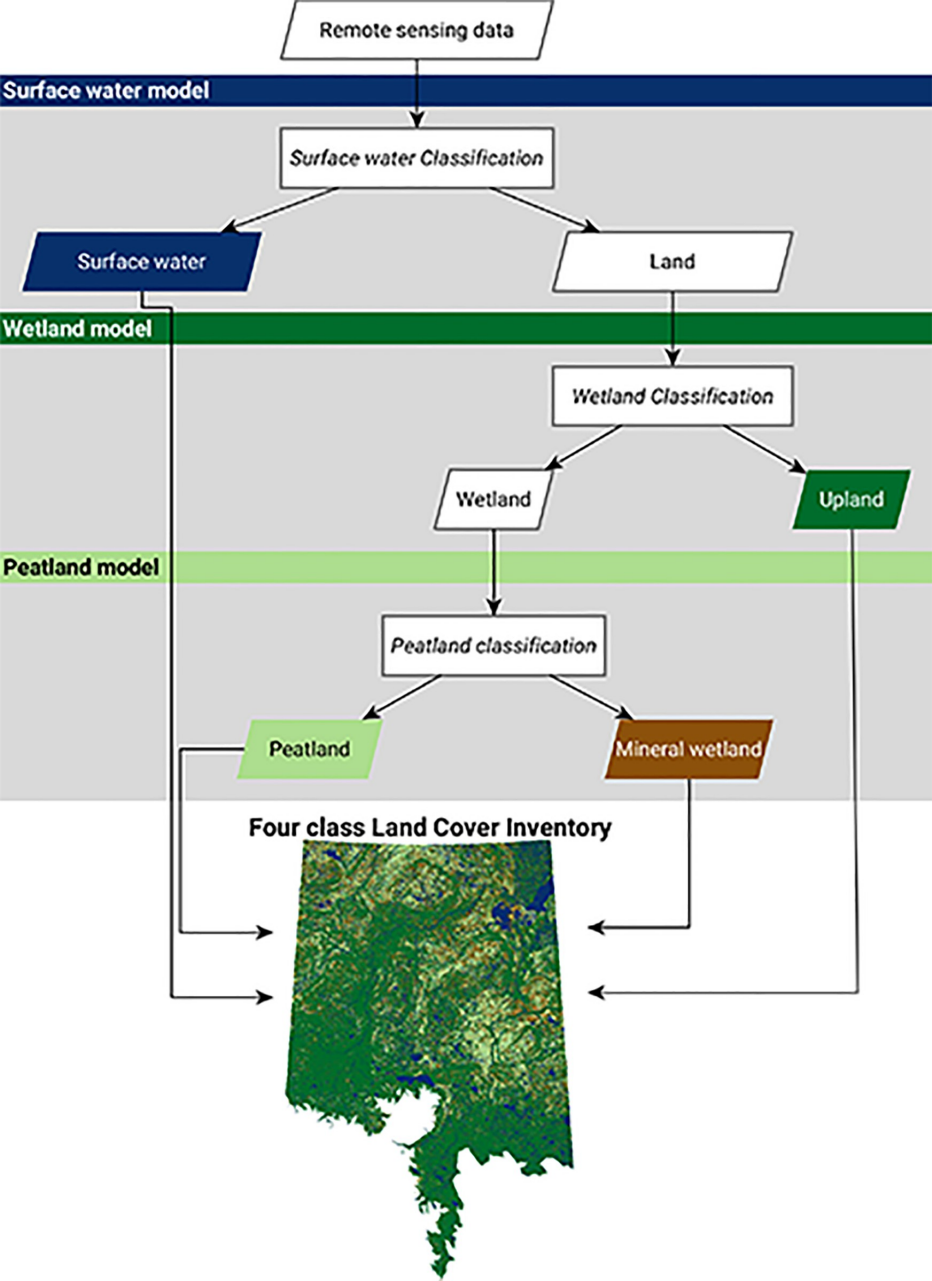
Flow chart of binary land cover classifications combining into a “traditional” four class landcover inventory. The ABMI has given permission to publish this image under a CC BY 4.0 license.

The prediction of peatland occurrence can be combined with other probabilistic classifications of landcover type [67, 68] to generate a “traditional” landcover map as seen in Fig 7. Individually creating binary landcover class splits (decision trees), as demonstrated in Fig 8, can be more advantageous than classification of multiple landcover types in one layer. Each split in the landcover tree (Fig 8) may need its own unique set of input geospatial variables. For example, when distinguishing water from land, SAR backscatter (VH) and optical-based wetness indices (NDWI) are typically of very high importance [68, 73]. On the other hand, VH and NDWI were not shown to be important in the peatland classification explored in this paper. For each step in the tree, a full analysis of all Earth observation variables and their relation to the binary landcover split should be studied. This creates a data-driven approach to the production of landcover inventories where each class split can be optimized, and model uncertainties at each class split can be explored. One major drawback of this approach is that errors at the top of the tree cannot be fixed further down. For example, if a peatland pixel is temporarily flooded and misclassified as surface water in the first split, this error will carry down through the remainder of the classification tree. Although this error could be fixed in the future with time-series optical data.

The data exploration and variable selection results, along with the model optimization (Supporting information) demonstrate that these are crucial steps when performing any kind of machine learning landcover classification. Fig 3 demonstrates that most of the remote sensing variables showed very little difference between the peatland and mineral wetland classes. Using these optimization and variable selection methods, we were able to bring the vast amounts of remote sensing data available down to six meaningful, uncorrelated input modelling variables. This process yielded input variables from three different earth observation data sources–DEMs, optical images, and SAR–indicating the importance of multiple data sources for landcover and wetland mapping [21]. Iteratively modifying parameters in the BRT model (Supporting information) allowed us to improve the overall accuracy of the classification by approximately 2%. The number of variables and number of training points employed was shown to have the largest effect on model performance. Other studies [74–76] have also highlighted the importance of variable selection, low variable multicollinearity, minimized spatial autocorrelation, and number of training sampling for machine learning wetland predictions.

The individual aspects of peatland mapping presented in this study, such as machine learning and high-resolution, large-scale prediction, are not novel on their own. For example, [15, 40] have used machine learning in the form of BRT and RandomForest models to predict peatland-occurrence in Canada. Both [12, 15] have predicted peatland occurrence across Canada, while [77] has shown that high resolution (2 m) mapping of wetlands in Alberta is feasible. The novelty of the current work lies in its integration of all these various techniques for peatland mapping, while providing a data-driven framework in which large-scale, high-resolution landcover inventories can be built from a vast numbers of Earth observation input variables. Additional novelty lies in the fact that this product can be generated with fully open-source Earth observation data, and processing software. Sentinel-1 and -2 data can be accessed and processed freely on the GEE platform. Although the LiDAR DEM used in this study is not open access, Table 3 shows us that an SRTM DEM, which is itself freely available on GEE, can be just as effective as a LiDAR DEM for peatland mapping. Finally, the training data used for this study (i.e., the ABMI 3x7 plots) is also open-access and can be downloaded on the ABMI’s website. The open-access nature of this framework makes large-scale landcover mapping accessible to many different users irrespective of budgetary constraints. With regard to peatlands and their importance for managing carbon budgets and meeting national or international emission goals, this approach is a solid first step toward a practical, scalable, and repeatable methodology for supporting long-term monitoring and management of natural resources.

While this methodology does produce spatially-consistent, large-scale, high-resolution classifications of landcover type, the actual accuracies of the classifications can be relatively low when attempting to differentiate structurally similar classes (e.g., wooded peatland vs. swamp, graminoid fen vs. marsh). This is due to the fact that traditional Earth observation data can show very little difference between classes as seen in Fig 3. To improve the accuracies of the peatland/mineral wetland model or any other land cover model one can either: 1) improve the input data or 2) improve the machine learning model.

One anticipated improvement in Earth observation data is to use bottom-of-atmosphere S2 data which could allow more refined mapping of broad vegetation types (we expect this to be available in Alberta later in 2019). Another improvement is to add time-series information to S1 and S2 data. Ideally each S1 and S2 variable could have a median, minimum, maximum, and standard deviation value which would increase the number of available modelling variables from 17 to 68 and perhaps enable greater capacity for separating often confused classes. In this study we attempted to use time-series S2 data but many pixels were limited to a single observation due to persistent cloud cover. Now that S2 has two operational satellites the potential for differentiating wetlands with seasonal cycles becomes a real possibility for future peatland/wetland classifications. L-band SAR could also be a potential improvement due to its ability to monitor water flow beneath peat accumulations [78]. More DEM-derived variables could also be generated such as different window sizes of TPI and other terrain metrics such as Valley Bottom Flatness Index, Mid Slope Position, and terrain ruggedness, among others. Accuracies could also be increased with better machine learning techniques and algorithms. Random Forest or Support Vector Machine (SVM) models may produce slightly better results as seen in [79], but initial tests in our work have shown very little difference between different models given the same input data. In fact, gradient boosting, of which BRT is grouped into, is the most common method of “shallow learning” in machine learning competitions and has been shown to outperform SVM and RandomForest algorithms in most competitions [80]. Since computing power is becoming less of a limiting factor it may actually be best to use ensemble models or model stacking to achieve high model accuracies [81]. More work is needed in comparing BRT, Random Forest, and SVM methods and determining the ideal situations for each, although the input and training data may end up having the greatest influence on model accuracy. Deep learning Convolutional Neural Networks such as TensorFlow may be a substantial upgrade for remote sensing machine learning algorithms [82–84]. The application of deep learning is relatively untested for traditional pixel-based classifications but novel applications of this technology will provide an exciting future for data-driven machine learning landcover classifications. The combination of pixel-based and object-based classification [85] may be a potential alternative method to increase accuracy since many pixel-based classifications possess a “salt and pepper” noise pattern. The incorporation of object-based classification may be able to smooth these patterns out into areas of continuous landcover classes which will better match the photo-interpreted training data.

Given the ease of accessing large areas of high-resolution satellite data in GEE, it is completely feasible to apply this modelling framework over larger areas such as all of Alberta or the Boreal Forest Region of Canada–thereby supporting a regional to national knowledge base regarding peatland location and extent. The main limitation of a national-scale application is the lack of reliable and accurate open-access training data and comprehensive land use (human footprint) information outside of Alberta. Indeed, in light of the current trend toward open-access satellite data and open-access machine learning libraries, the principal challenge for large-scale wetland and landcover mapping initiatives appears to be the limited availability of high quality, open-access training data, which itself will always require detailed sources of information, such as that from manual photo-interpretation or field work.

## Conclusion

The global importance of peatlands as carbon sinks and as wetland ecosystems providing a host of ecosystem services provides an impetus for accurate and comprehensive mapping strategies. Practitioners affiliated with organizations like the International Peatland Society and the Boreal Research Institute of Alberta require accurate high-resolution maps to assist with site prioritization and development of management practices. Knowing the location, extent, and quantity of peatlands is of fundamental importance to understanding carbon storage potential, and a prerequisite to any restoration and reclamation efforts.

Our study demonstrates the application of a framework which uses cloud-based access of open-access satellite data sets, in the form of Google Earth Engine and open-access machine learning models within R Statistical Software, for large-scale, high-resolution mapping of northern (subarctic, boreal, temperate) peatlands. Applying this model over our entire study area resulted in an 86% overall accuracy when distinguishing peatlands versus non-peatlands, and an overall accuracy of 69% when differentiating peatlands from mineral wetlands. Variable selection, data exploration, and model optimization were shown to be very important, highlighting the need for data-driven decisions for model parameterization such as input variable intra-correlation, number of modelling variables, and number of training samples. The approach described here brings us closer to more accurate understandings of peatland distribution, and therefore to better management and effective monitoring as our landscape and climate continue to change.

While the model proved to be relatively successful, there are still many improvements that can be made such as the inclusion of multi-temporal Earth observation inputs, deep learning algorithms, and the fusion of pixel- and object-based approaches to classification. Nevertheless, this study offers a framework for leveraging large amounts of open-access earth observation data to produce binary landcover classifications at regional to national scales. Advances in cloud computing, open-access data, and machine learning technologies will push forward the development of large-scale landcover inventories by expanding the numbers of geospatial data users, fostering increased collaboration, and providing a means of meeting current and new challenges in large-area mapping and monitoring.

## Supporting information

S1 File The supporting information contains a model optimization experiment, Google Earth Engine Code, and R code. (DOCX)

S1 Data The accuracy of the peatland classification with varying: LR, TC, number of variables, and number of training points. The ABMI has given permission to publish this image under a CC BY 4.0 license.
(TIFF)
