# Supplementary material for: Large-scale probabilistic identification of boreal peatlands using Google Earth Engine, open-access satellite data, and machine learning
Source: PLoS One. 2019 Jun 17;14(6):e0218165. doi: 10.1371/journal.pone.0218165 (PMC6576777; doi:10.1371/journal.pone.0218165)
Supplement: S1 File — (DOCX) [file pone.0218165.s001.docx]

# Supporting Information

## Model optimization

S1 Fig. shows the results of our model parameter optimization tests. Varying the learning rate showed no trend in terms of accuracy, and we therefore chose a 0.005 learning rate which is common in other BRT studies (Parisien et al., 2011; Thompson et al., 2016). Accuracy increased by 0.5% when increasing tree complexity from 2 to 8, with a subsequent leveling-off at tree complexities of 9 and 10. A tree complexity of 8 was selected for modelling. Altering the number of variables from 2 to 8 showed an increasing trend until the inclusion of the eighth variable. Six variables were chosen for modelling since a more simplistic model is preferred and the increase in accuracy moving from six to seven variables was very small. Lastly, accuracy was seen to increase logistically with an increased number of training points. While 91,347 training points would yield the highest accuracy, this would dramatically increase the processing time to predict peatland-occurrence across the entire BNR (3.9 billion pixels). Therefore, a total of 6,497 training samples was chosen for modelling since this was the number where the increase in accuracy starts to level off. In summary, we chose to optimize our BRT model with a learning rate of 0.005, tree complexity of 8, six variables (REIP, PC1, TWI, TPI, NDPOL, ARI), and 6,497 training samples. Code for this model optimization can be found in the following GitHub repository (<https://github.com/evan-delancey/PeatlandProbability>).

**S1 Data. The accuracy of the peatland classification with varying: LR, TC, number of variables, and number of training points. The ABMI has given permission to publish this image under a CC BY 4.0 license.**

## Google Earth Engine code

The following code was used to collect the Sentinel-1, -2, and SRTM input modelling variables. The code is written in JavaScript and can be pasted into the code editor (https://code.earthengine.google.com/ ). The code can also be seen in the following GitHub repository (https://github.com/evan-delancey/GoogleEarthEngine/blob/master/PeatlandProbabilityInputs.js) and updated, more recent versions of the code can be seen here (https://github.com/evan-delancey/GoogleEarthEngine).

| /////////////////////////////////////////////////////////////////////  //PeatlandProbabilityInputs.js - GEE script /  //Written by: /  ////Evan R. DeLancey /  ////GIS Land-use analyst Alberta Biodiversity Monitoring Institute /  ////2017-08-24 /  ////edelance@ualberta.ca /  /////////////////////////////////////////////////////////////////////  ///////////////////////////////////////////////////////////////////////////////  //Define Alberta shape and projection file  ///////////////////////////////////////////////////////////////////////////////  **var** PrjFile **=** ee**.**Image**(**'users/abmigc/ProcessingUnits'**);**  **var** prj **=** PrjFile**.**projection**();**  **var** AB **=** ee**.**FeatureCollection**(**'users/abmigc/Alberta_prjGEE'**);**  ///////////////////////////////////////////////////////////////////////////////  //END  ///////////////////////////////////////////////////////////////////////////////  ///////////////////////////////////////////////////////////////////////////////  //Define Sentinel-1 functions  ///////////////////////////////////////////////////////////////////////////////  //Angle correction and add NDPOL  **function** S1Prep**(image)** **{**  **var** first **=** **image.**addBands**(image.select(**'VV'**).**subtract**(image.select(**'angle'**).**multiply**(**Math**.**PI**/**180.0**).**cos**().**log10**().**multiply**(**10.0**)).**rename**(**'VV_Gamma'**));**  **var** second **=** first**.**addBands**(image.select(**'VH'**).**subtract**(image.select(**'angle'**).**multiply**(**Math**.**PI**/**180.0**).**cos**().**log10**().**multiply**(**10.0**)).**rename**(**'VH_Gamma'**));**  **return** second**.**addBands**(**second**.**normalizedDifference**([**'VH_Gamma'**,** 'VV_Gamma'**]).**rename**(**'NDPOL'**));**  **}**  //Sigma Lee filter  **function** toNatural**(**img**)** **{**  **return** ee**.**Image**(**10.0**).**pow**(**img**.select(**0**).**divide**(**10.0**));**  **}**  **function** toDB**(**img**)** **{**  **return** ee**.**Image**(**img**).**log10**().**multiply**(**10.0**);**  **}**  // The RL speckle filter from https://code.earthengine.google.com/2ef38463ebaf5ae133a478f173fd0ab5  // by Guido Lemoine  **function** RefinedLee**(**img**)** **{**  // img must be in natural units, i.e. not in dB!  // Set up 3x3 kernels  **var** weights3 **=** ee**.**List**.**repeat**(**ee**.**List**.**repeat**(**1**,**3**),**3**);**  **var** kernel3 **=** ee**.**Kernel**.**fixed**(**3**,**3**,** weights3**,** 1**,** 1**,** **false);**  **var** mean3 **=** img**.**reduceNeighborhood**(**ee**.**Reducer**.**mean**(),** kernel3**);**  **var** variance3 **=** img**.**reduceNeighborhood**(**ee**.**Reducer**.**variance**(),** kernel3**);**  // Use a sample of the 3x3 windows inside a 7x7 windows to determine gradients and directions  **var** sample_weights **=** ee**.**List**([[**0**,**0**,**0**,**0**,**0**,**0**,**0**],** **[**0**,**1**,**0**,**1**,**0**,**1**,**0**],[**0**,**0**,**0**,**0**,**0**,**0**,**0**],** **[**0**,**1**,**0**,**1**,**0**,**1**,**0**],** **[**0**,**0**,**0**,**0**,**0**,**0**,**0**],** **[**0**,**1**,**0**,**1**,**0**,**1**,**0**],[**0**,**0**,**0**,**0**,**0**,**0**,**0**]]);**  **var** sample_kernel **=** ee**.**Kernel**.**fixed**(**7**,**7**,** sample_weights**,** 3**,**3**,** **false);**  // Calculate mean and variance for the sampled windows and store as 9 bands  **var** sample_mean **=** mean3**.**neighborhoodToBands**(**sample_kernel**);**  **var** sample_var **=** variance3**.**neighborhoodToBands**(**sample_kernel**);**  // Determine the 4 gradients for the sampled windows  **var** gradients **=** sample_mean**.select(**1**).**subtract**(**sample_mean**.select(**7**)).**abs**();**  gradients **=** gradients**.**addBands**(**sample_mean**.select(**6**).**subtract**(**sample_mean**.select(**2**)).**abs**());**  gradients **=** gradients**.**addBands**(**sample_mean**.select(**3**).**subtract**(**sample_mean**.select(**5**)).**abs**());**  gradients **=** gradients**.**addBands**(**sample_mean**.select(**0**).**subtract**(**sample_mean**.select(**8**)).**abs**());**  // And find the maximum gradient amongst gradient bands  **var** max_gradient **=** gradients**.**reduce**(**ee**.**Reducer**.**max**());**  // Create a mask for band pixels that are the maximum gradient  **var** gradmask **=** gradients**.**eq**(**max_gradient**);**  // duplicate gradmask bands: each gradient represents 2 directions  gradmask **=** gradmask**.**addBands**(**gradmask**);**  // Determine the 8 directions  **var** directions **=** sample_mean**.select(**1**).**subtract**(**sample_mean**.select(**4**)).**gt**(**sample_mean**.select(**4**).**subtract**(**sample_mean**.select(**7**))).**multiply**(**1**);**  directions **=** directions**.**addBands**(**sample_mean**.select(**6**).**subtract**(**sample_mean**.select(**4**)).**gt**(**sample_mean**.select(**4**).**subtract**(**sample_mean**.select(**2**))).**multiply**(**2**));**  directions **=** directions**.**addBands**(**sample_mean**.select(**3**).**subtract**(**sample_mean**.select(**4**)).**gt**(**sample_mean**.select(**4**).**subtract**(**sample_mean**.select(**5**))).**multiply**(**3**));**  directions **=** directions**.**addBands**(**sample_mean**.select(**0**).**subtract**(**sample_mean**.select(**4**)).**gt**(**sample_mean**.select(**4**).**subtract**(**sample_mean**.select(**8**))).**multiply**(**4**));**  // The next 4 are the not() of the previous 4  directions **=** directions**.**addBands**(**directions**.select(**0**).**not**().**multiply**(**5**));**  directions **=** directions**.**addBands**(**directions**.select(**1**).**not**().**multiply**(**6**));**  directions **=** directions**.**addBands**(**directions**.select(**2**).**not**().**multiply**(**7**));**  directions **=** directions**.**addBands**(**directions**.select(**3**).**not**().**multiply**(**8**));**  // Mask all values that are not 1-8  directions **=** directions**.**updateMask**(**gradmask**);**  // "collapse" the stack into a singe band image (due to masking, each pixel has just one value (1-8) in it's directional band, and is otherwise masked)  directions **=** directions**.**reduce**(**ee**.**Reducer**.**sum**());**  //var pal = ['ffffff','ff0000','ffff00', '00ff00', '00ffff', '0000ff', 'ff00ff', '000000'];  //Map.addLayer(directions.reduce(ee.Reducer.sum()), {min:1, max:8, palette: pal}, 'Directions', false);  **var** sample_stats **=** sample_var**.**divide**(**sample_mean**.**multiply**(**sample_mean**));**  // Calculate localNoiseVariance  **var** sigmaV **=** sample_stats**.**toArray**().**arraySort**().**arraySlice**(**0**,**0**,**5**).**arrayReduce**(**ee**.**Reducer**.**mean**(),** **[**0**]);**  // Set up the 7*7 kernels for directional statistics  **var** rect_weights **=** ee**.**List**.**repeat**(**ee**.**List**.**repeat**(**0**,**7**),**3**).**cat**(**ee**.**List**.**repeat**(**ee**.**List**.**repeat**(**1**,**7**),**4**));**  **var** diag_weights **=** ee**.**List**([[**1**,**0**,**0**,**0**,**0**,**0**,**0**],** **[**1**,**1**,**0**,**0**,**0**,**0**,**0**],** **[**1**,**1**,**1**,**0**,**0**,**0**,**0**],**  **[**1**,**1**,**1**,**1**,**0**,**0**,**0**],** **[**1**,**1**,**1**,**1**,**1**,**0**,**0**],** **[**1**,**1**,**1**,**1**,**1**,**1**,**0**],** **[**1**,**1**,**1**,**1**,**1**,**1**,**1**]]);**  **var** rect_kernel **=** ee**.**Kernel**.**fixed**(**7**,**7**,** rect_weights**,** 3**,** 3**,** **false);**  **var** diag_kernel **=** ee**.**Kernel**.**fixed**(**7**,**7**,** diag_weights**,** 3**,** 3**,** **false);**  // Create stacks for mean and variance using the original kernels. Mask with relevant direction.  **var** dir_mean **=** img**.**reduceNeighborhood**(**ee**.**Reducer**.**mean**(),** rect_kernel**).**updateMask**(**directions**.**eq**(**1**));**  **var** dir_var **=** img**.**reduceNeighborhood**(**ee**.**Reducer**.**variance**(),** rect_kernel**).**updateMask**(**directions**.**eq**(**1**));**  dir_mean **=** dir_mean**.**addBands**(**img**.**reduceNeighborhood**(**ee**.**Reducer**.**mean**(),** diag_kernel**).**updateMask**(**directions**.**eq**(**2**)));**  dir_var **=** dir_var**.**addBands**(**img**.**reduceNeighborhood**(**ee**.**Reducer**.**variance**(),** diag_kernel**).**updateMask**(**directions**.**eq**(**2**)));**  // and add the bands for rotated kernels  **for** **(var** i**=**1**;** i**<**4**;** i**++)** **{**  dir_mean **=** dir_mean**.**addBands**(**img**.**reduceNeighborhood**(**ee**.**Reducer**.**mean**(),** rect_kernel**.**rotate**(**i**)).**updateMask**(**directions**.**eq**(**2*****i**+**1**)));**  dir_var **=** dir_var**.**addBands**(**img**.**reduceNeighborhood**(**ee**.**Reducer**.**variance**(),** rect_kernel**.**rotate**(**i**)).**updateMask**(**directions**.**eq**(**2*****i**+**1**)));**  dir_mean **=** dir_mean**.**addBands**(**img**.**reduceNeighborhood**(**ee**.**Reducer**.**mean**(),** diag_kernel**.**rotate**(**i**)).**updateMask**(**directions**.**eq**(**2*****i**+**2**)));**  dir_var **=** dir_var**.**addBands**(**img**.**reduceNeighborhood**(**ee**.**Reducer**.**variance**(),** diag_kernel**.**rotate**(**i**)).**updateMask**(**directions**.**eq**(**2*****i**+**2**)));**  **}**  // "collapse" the stack into a single band image (due to masking, each pixel has just one value in it's directional band, and is otherwise masked)  dir_mean **=** dir_mean**.**reduce**(**ee**.**Reducer**.**sum**());**  dir_var **=** dir_var**.**reduce**(**ee**.**Reducer**.**sum**());**  // A finally generate the filtered value  **var** varX **=** dir_var**.**subtract**(**dir_mean**.**multiply**(**dir_mean**).**multiply**(**sigmaV**)).**divide**(**sigmaV**.**add**(**1.0**));**  **var** b **=** varX**.**divide**(**dir_var**);**  **var** result **=** dir_mean**.**add**(**b**.**multiply**(**img**.**subtract**(**dir_mean**)));**  **return(**result**.**arrayFlatten**([[**'sum'**]]));**  **}**  //angle masking  **var** maskAng1 **=** **function(image)** **{**  **var** ang **=** **image.select([**'angle'**]);**  **return** **image.**updateMask**(**ang**.**gt**(**30.53993**));**  **};**  **var** maskAng2 **=** **function(image)** **{**  **var** ang **=** **image.select([**'angle'**]);**  **return** **image.**updateMask**(**ang**.**lt**(**45.53993**));**  **};**  **var** maskAng3 **=** **function(image)** **{**  **var** ang **=** **image.select([**'angle'**]);**  **return** **image.**updateMask**(**ang**.**gt**(**30.63993**));**  **};**  **var** maskAng4 **=** **function(image)** **{**  **var** ang **=** **image.select([**'angle'**]);**  **return** **image.**updateMask**(**ang**.**lt**(**44.73993**));**  **};**  ///////////////////////////////////////////////////////////////////////////////  //END  ///////////////////////////////////////////////////////////////////////////////    ///////////////////////////////////////////////////////////////////  // START Get Sentinel-1 image stack for 2016 and 2017  ///////////////////////////////////////////////////////////////////  **var** s1_1 **=** ee**.**ImageCollection**(**'COPERNICUS/S1_GRD'**)**  **.**filterBounds**(**AB**)**  **.**filterDate**(**'2016-07-01'**,** '2016-07-31'**)**  **.**filterMetadata**(**'transmitterReceiverPolarisation'**,** 'equals'**,** **[**'VV'**,** 'VH'**])**  **.**filterMetadata**(**'resolution_meters'**,** 'equals' **,** 10**);**  **var** s1_1 **=** s1_1**.**map**(**maskAng1**);**  **var** s1_1 **=** s1_1**.**map**(**maskAng2**);**  **var** s1_2 **=** ee**.**ImageCollection**(**'COPERNICUS/S1_GRD'**)**  **.**filterBounds**(**AB**)**  **.**filterDate**(**'2016-08-01'**,** '2016-08-10'**)**  **.**filterMetadata**(**'transmitterReceiverPolarisation'**,** 'equals'**,** **[**'VV'**,** 'VH'**])**  **.**filterMetadata**(**'resolution_meters'**,** 'equals' **,** 10**);**  **var** s1_2 **=** s1_2**.**map**(**maskAng1**);**  **var** s1_2 **=** s1_2**.**map**(**maskAng2**);**  **var** s1_2b **=** ee**.**ImageCollection**(**'COPERNICUS/S1_GRD'**)**  **.**filterBounds**(**AB**)**  **.**filterDate**(**'2016-08-11'**,** '2016-08-31'**)**  **.**filterMetadata**(**'transmitterReceiverPolarisation'**,** 'equals'**,** **[**'VV'**,** 'VH'**])**  **.**filterMetadata**(**'resolution_meters'**,** 'equals' **,** 10**);**  **var** s1_2b **=** s1_2b**.**map**(**maskAng1**);**  **var** s1_2b **=** s1_2b**.**map**(**maskAng2**);**  **var** s1_3 **=** ee**.**ImageCollection**(**'COPERNICUS/S1_GRD'**)**  **.**filterBounds**(**AB**)**  **.**filterDate**(**'2017-05-15'**,** '2017-08-31'**)**  **.**filterMetadata**(**'transmitterReceiverPolarisation'**,** 'equals'**,** **[**'VV'**,** 'VH'**])**  **.**filterMetadata**(**'resolution_meters'**,** 'equals' **,** 10**);**  **var** s1_3 **=** s1_3**.**map**(**maskAng3**);**  **var** s1_3 **=** s1_3**.**map**(**maskAng4**);**  **var** s1 **=** ee**.**ImageCollection**(**s1_1**.**merge**(**s1_2**));**  **var** s1 **=** ee**.**ImageCollection**(**s1**.**merge**(**s1_3**));**  **var** s1 **=** ee**.**ImageCollection**(**s1**.**merge**(**s1_2b**));**  ///////////////////////////////////////////////////////////////////////////////  //END  ///////////////////////////////////////////////////////////////////////////////  ///////////////////////////////////////////////////////////////////////////////  //Map functions over Sentinel-1 image stack  ///////////////////////////////////////////////////////////////////////////////  **var** s1 **=** s1**.**map**(**S1Prep**);**  **var** VH **=** s1**.select([**'VH_Gamma'**]);**  **var** NDPOL **=** s1**.select([**'NDPOL'**]);**  **var** VH **=** VH**.**map**(**toNatural**);**  **var** VH **=** VH**.**map**(**RefinedLee**);**  **var** VH **=** VH**.**map**(**toDB**);**  ///////////////////////////////////////////////////////////////////////////////  //END  ///////////////////////////////////////////////////////////////////////////////  ///////////////////////////////////////////////////////////////////////////////  //Spatially filter NDPOL  ///////////////////////////////////////////////////////////////////////////////  **var** boxcar **=** ee**.**Kernel**.**circle**({**  radius**:** 3**,** units**:** 'pixels'**,** normalize**:** **true**  **});**  **function** fltr**(image)** **{**  **return** **image.**convolve**(**boxcar**);**  **}**  **var** NDPOL **=** NDPOL**.**map**(**fltr**);**  ///////////////////////////////////////////////////////////////////////////////  //END  ///////////////////////////////////////////////////////////////////////////////  ///////////////////////////////////////////////////////////////////////////////  //Take temporal mean of picel stack and add layers to map  ///////////////////////////////////////////////////////////////////////////////  **var** VH **=** VH**.**mean**();**  **var** NDPOL **=** NDPOL**.**mean**();**  Map**.**addLayer**(**VH**,** **{**min**:-**28**,** max**:-**8**},** 'VH'**);**  Map**.**addLayer**(**NDPOL**,** **{**min**:**0**,** max**:**0.5**},** 'NPOL'**);**  ///////////////////////////////////////////////////////////////////////////////  //END  ///////////////////////////////////////////////////////////////////////////////  ///////////////////////////////////////////////////////////////////////////////  //Reproject and clip Sentinel-1 variables to Alberta  ///////////////////////////////////////////////////////////////////////////////  **var** VH **=** VH**.**reproject**(**prj**,** **null,** 10**).**clip**(**AB**);**  **var** NDPOL **=** NDPOL**.**reproject**(**prj**,** **null,** 10**).**clip**(**AB**);**  ///////////////////////////////////////////////////////////////////////////////  //END  ///////////////////////////////////////////////////////////////////////////////  //////////////////////////////////////////////////////////////////  //Export Sentinel-1 variables to drive  /////////////////////////////////////////////////////////////////  Export**.image.**toDrive**({**  **image:** VH**,**  description**:** 'VH'**,**  scale**:** 10**,**  region**:** AB**,**  maxPixels**:** 3E10  **});**  Export**.image.**toDrive**({**  **image:** NDPOL**,**  description**:** 'NDPOL'**,**  scale**:** 10**,**  region**:** AB**,**  maxPixels**:** 3E10  **});**  ///////////////////////////////////////////////////////////////  // END  ///////////////////////////////////////////////////////////////  ///////////////////////////////////////////////////////////////  // Sentinel-2 functions  ///////////////////////////////////////////////////////////////  //mask clouds  **function** maskCloud**(image)** **{**  **var** QA60 **=** **image.select([**'QA60'**]);**  **var** B1 **=** **image.select([**'B1'**]).**gt**(**1500**);**  **var** mask1 **=** **image.**updateMask**(**QA60**.**lt**(**1**));**  **return** mask1**.**updateMask**(**B1**.**lt**(**1**));**  **}**  //add indices  **function** addIndices**(image)** **{**  **var** a **=** **image.**addBands**(image.**normalizedDifference**([**'B8'**,** 'B4'**]).**rename**(**'NDVI'**));**  **var** b **=** a**.**addBands**(**a**.**normalizedDifference**([**'B3'**,** 'B8'**]).**rename**(**'NDWI'**));**  **var** c **=** b**.**addBands**(**b**.**expression**(**  '(B8 / B2) - (B8 / B3)'**,** **{**  'B8'**:** **image.select([**'B8'**]),**  'B2'**:** **image.select([**'B2'**]),**  'B3'**:** **image.select([**'B3'**])**  **}**  **).**rename**(**'ARI'**));**  **var** d **=** c**.**addBands**(**c**.**expression**(**  '(B4 - B2) / B5'**,** **{**  'B4'**:** **image.select([**'B4'**]),**  'B2'**:** **image.select([**'B2'**]),**  'B5'**:** **image.select([**'B5'**])**  **}**  **).**rename**(**'PSRI'**));**  **return** d**.**addBands**(**d**.**expression**(**  '705 + 35*((((RED + RE3)/2) - RE1) / (RE2 - RE1))'**,** **{**  'RE1'**:** **image.select([**'B5'**]),**  'RE2'**:** **image.select([**'B6'**]),**  'RE3'**:** **image.select([**'B7'**]),**  'RED' **:** **image.select([**'B4'**])**  **}**  **).**rename**(**'REIP'**));**  **}**  ///////////////////////////////////////////////////////////////  //END  ///////////////////////////////////////////////////////////////  ///////////////////////////////////////////////////////////////  //Get Sentinel-1 image stack  ///////////////////////////////////////////////////////////////  **var** S2 **=** ee**.**ImageCollection**(**'COPERNICUS/S2'**)**  **.**filterDate**(**'2016-05-15'**,** '2016-08-31'**)**  **.**filterBounds**(**AB**);**  **var** S2_1 **=** ee**.**ImageCollection**(**'COPERNICUS/S2'**)**  **.**filterDate**(**'2017-05-15'**,** '2017-08-31'**)**  **.**filterBounds**(**AB**);**  **var** S2 **=** ee**.**ImageCollection**(**S2**.**merge**(**S2_1**));**  ///////////////////////////////////////////////////////////////  //END  ///////////////////////////////////////////////////////////////  ///////////////////////////////////////////////////////////////  //Map functions over image stack  ///////////////////////////////////////////////////////////////  **var** S2 **=** S2**.**map**(**maskCloud**);**  **var** S2 **=** S2**.**map**(**addIndices**);**  ///////////////////////////////////////////////////////////////  //END  ///////////////////////////////////////////////////////////////  ///////////////////////////////////////////////////////////////  //Get median value of each index and band and add to map  ///////////////////////////////////////////////////////////////  **var** B2 **=** S2**.select([**'B2'**]).**median**();**  **var** B3 **=** S2**.select([**'B3'**]).**median**();**  **var** B4 **=** S2**.select([**'B4'**]).**median**();**  **var** B8 **=** S2**.select([**'B8'**]).**median**();**  **var** NDVI **=** S2**.select([**'NDVI'**]).**median**();**  **var** NDWI **=** S2**.select([**'NDWI'**]).**median**();**  **var** ARI **=** S2**.select([**'ARI'**]).**median**();**  **var** PSRI **=** S2**.select([**'PSRI'**]).**median**();**  **var** REIP **=** S2**.select([**'REIP'**]).**median**();**  **var** colorbrewer **=** require**(**'users/gena/packages:colorbrewer'**);**  Map**.**addLayer**(**NDVI**,** **{**min**:-**0.5**,** max**:**0.9**,** palette**:** colorbrewer**.**Palettes**.**RdYlGn**[**11**]},** 'NDVI'**);**  Map**.**addLayer**(**NDWI**,** **{**min**:-**1**,** max**:**1**,** palette**:** colorbrewer**.**Palettes**.**Blues**[**9**]},** 'NDWI'**);**  Map**.**addLayer**(**ARI**,** **{**min**:-**1**,** max**:**0.3**,** palette**:** colorbrewer**.**Palettes**.**PRGn**[**11**]},** 'ARI'**);**  Map**.**addLayer**(**PSRI**,** **{**min**:-**1**,** max**:**1**,** palette**:** colorbrewer**.**Palettes**.**PRGn**[**11**]},** 'PSRI'**);**  Map**.**addLayer**(**REIP**,** **{**min**:**715**,** max**:**730**,** palette**:** colorbrewer**.**Palettes**.**PRGn**[**11**]},** 'REIP'**);**  ///////////////////////////////////////////////////////////////  //END  ///////////////////////////////////////////////////////////////  ///////////////////////////////////////////////////////////////  //Reproject indices and clip to Alberta  ///////////////////////////////////////////////////////////////  **var** B2 **=** B2**.**reproject**(**prj**,** **null,** 10**).**clip**(**AB**);**  **var** B3 **=** B3**.**reproject**(**prj**,** **null,** 10**).**clip**(**AB**);**  **var** B4 **=** B4**.**reproject**(**prj**,** **null,** 10**).**clip**(**AB**);**  **var** B8 **=** B8**.**reproject**(**prj**,** **null,** 10**).**clip**(**AB**);**  **var** NDVI **=** NDVI**.**reproject**(**prj**,** **null,** 10**).**clip**(**AB**);**  **var** NDWI **=** NDWI**.**reproject**(**prj**,** **null,** 10**).**clip**(**AB**);**  **var** ARI **=** ARI**.**reproject**(**prj**,** **null,** 10**).**clip**(**AB**);**  **var** PSRI **=** PSRI**.**reproject**(**prj**,** **null,** 10**).**clip**(**AB**);**  **var** REIP **=** REIP**.**reproject**(**prj**,** **null,** 10**).**clip**(**AB**);**  ///////////////////////////////////////////////////////////////  //END  ///////////////////////////////////////////////////////////////  ///////////////////////////////////////////////////////////////  //Export Sentinel-2 indices to drive  ///////////////////////////////////////////////////////////////  Export**.image.**toDrive**({**  **image:** B2**,**  description**:** 'B2'**,**  scale**:** 10**,**  region**:** AB**,**  maxPixels**:** 10E10  **});**  Export**.image.**toDrive**({**  **image:** B3**,**  description**:** 'B3'**,**  scale**:** 10**,**  region**:** AB**,**  maxPixels**:** 10E10  **});**  Export**.image.**toDrive**({**  **image:** B4**,**  description**:** 'B4'**,**  scale**:** 10**,**  region**:** AB**,**  maxPixels**:** 10E10  **});**  Export**.image.**toDrive**({**  **image:** B8**,**  description**:** 'B8'**,**  scale**:** 10**,**  region**:** AB**,**  maxPixels**:** 10E10  **});**  Export**.image.**toDrive**({**  **image:** NDWI**,**  description**:** 'NDWI'**,**  scale**:** 10**,**  region**:** AB**,**  maxPixels**:** 10E10  **});**  Export**.image.**toDrive**({**  **image:** NDVI**,**  description**:** 'NDVI'**,**  scale**:** 10**,**  region**:** AB**,**  maxPixels**:** 10E10  **});**  Export**.image.**toDrive**({**  **image:** ARI**,**  description**:** 'ARI'**,**  scale**:** 10**,**  region**:** AB**,**  maxPixels**:** 10E10  **});**  Export**.image.**toDrive**({**  **image:** PSRI**,**  description**:** 'PSRI'**,**  scale**:** 10**,**  region**:** AB**,**  maxPixels**:** 10E10  **});**  Export**.image.**toDrive**({**  **image:** REIP**,**  description**:** 'REIP'**,**  scale**:** 10**,**  region**:** AB**,**  maxPixels**:** 10E10  **});**  ///////////////////////////////////////////////////////////////  //END  ///////////////////////////////////////////////////////////////  ///////////////////////////////////////////////////////////////  //Get SRTM DEM, smooth, and export to drive  ///////////////////////////////////////////////////////////////  **var** srtm **=** ee**.**Image**(**'USGS/SRTMGL1_003'**);**  **var** srtm **=** srtm**.**reproject**(**prj**,** **null,** 10**).**clip**(**AB**);**  **var** boxcar **=** ee**.**Kernel**.**circle**({**  radius**:** 7**,** units**:** 'pixels'**,** normalize**:** **true**  **});**  **var** srtm **=** srtm**.**convolve**(**boxcar**);**  Export**.image.**toDrive**({**  **image:** srtm**,**  description**:** 'SRTM'**,**  scale**:** 10**,**  region**:** AB**,**  maxPixels**:** 9E10  **});**  ///////////////////////////////////////////////////////////////  //END  ///////////////////////////////////////////////////////////////  ///////////////////////////////////////////////////////////////  //END script  /////////////////////////////////////////////////////////////// |
| --- |

## R Boosted Regression Tree code

This R code runs the methods described in the section “Wetland classification – machine learning algorithm and spatial prediction”. This code models peatland probability with reference to the ABMI plot data and then predicts across the entire study region. This code along with other model optimization code for this project can be seen in the following GitHub repository (https://github.com/evan-delancey/PeatlandProbability).

| ####################################################################  #------------------------------------------------------------------#  #------------------------------------------------------------------#  #Boreal Organic wetland probability #  #Filename: "Boreal_PeatlandProbability.R" #  #Written and developed by Evan R. DeLancey - GIS Land Use Analyst #  #Alberta Biodiversity Monitoring Institute, Jan, 30, 2018 #  #------------------------------------------------------------------#  #------------------------------------------------------------------#  ####################################################################  #Load libraries  library**(**raster**)**  library**(**rgdal**)**  library**(**ggplot2**)**  library**(**dplyr**)**  library**(**caret**)**  library**(**snow**)**  library**(**rgeos**)**  library**(**RPyGeo**)**  library**(**dismo**)**  library**(**gbm**)**  library**(**RStoolbox**)**  library**(**ggthemes**)**  tt1 **<-** Sys.time**()**  #location of input rasters  location **<-** "J:/LandCover/ProbabilityofWetArea/Boreal/processed"  #location of land and wetland shapefiles  location.training **<-** "J:/LandCover/OrganicWetlandProbability/Training"  #location of training points  location.pts **<-** "J:/LandCover/OrganicWetlandProbability/Training/ModelPts/Data375"  #location of outputs  outputs **<-** "J:/LandCover/OrganicWetlandProbability/OUTPUTS1"  #enter number of iteration of subsampling  iter **<-** 40  #set location of a temporary raster dump  #this can take up 100-300BG per run but is deleted after  rasterOptions**(**maxmemory **=** 1e**+**09,tmpdir **=** "J:/RtmpRasterDump"**)**  #DEFINE FUNCTIONS  ###################################################################  #------------------------------------------------------------------  #------------------------------------------------------------------  #1)multiplot function  multiplot **<-** **function(**..., plotlist**=NULL**, file, cols**=**1, layout**=NULL)** **{**  library**(**grid**)**  # Make a list from the ... arguments and plotlist  plots **<-** c**(**list**(**...**)**, plotlist**)**  numPlots **=** length**(**plots**)**  # If layout is NULL, then use 'cols' to determine layout  **if** **(**is.null**(**layout**))** **{**  # Make the panel  # ncol: Number of columns of plots  # nrow: Number of rows needed, calculated from # of cols  layout **<-** matrix**(**seq**(**1, cols ***** ceiling**(**numPlots**/**cols**))**,  ncol **=** cols, nrow **=** ceiling**(**numPlots**/**cols**))**  **}**  **if** **(**numPlots**==**1**)** **{**  print**(**plots**[[**1**]])**  **}** **else** **{**  # Set up the page  grid.newpage**()**  pushViewport**(**viewport**(**layout **=** grid.layout**(**nrow**(**layout**)**, ncol**(**layout**))))**  # Make each plot, in the correct location  **for** **(**i **in** 1**:**numPlots**)** **{**  # Get the i,j matrix positions of the regions that contain this subplot  matchidx **<-** as.data.frame**(**which**(**layout **==** i, arr.ind **=** **TRUE))**  print**(**plots**[[**i**]]**, vp **=** viewport**(**layout.pos.row **=** matchidx**$**row,  layout.pos.col **=** matchidx**$**col**))**  **}**  **}**  **}**  #----------------------------------------------------------------  #----------------------------------------------------------------  #################################################################  #Name vars and get min and max for response curves  ###################################################################  #------------------------------------------------------------------  #------------------------------------------------------------------  #set input varibles  tifs **<-** c**(**"ARI.tif", "NDPOL.tif", "PC1.tif", "REIP.tif", "TPI.tif", "TWI.tif"**)**  collumn.names **<-** c**(**"ARI", "NDPOL", "PC1", "REIP", "TPI", "TWI", "Owetland"**)**  bricknames **<-** c**(**"ARI", "NDPOL", "PC1", "REIP", "TPI", "TWI"**)**  fls **<-** tifs  setwd**(**location**)**  #extract min and max of all input rasters for response curves  r **<-** raster**(**fls**[**1**])**  min1 **<-** cellStats**(**r, 'min'**)**  max1 **<-** cellStats**(**r, 'max'**)**  r **<-** raster**(**fls**[**2**])**  min2 **<-** cellStats**(**r, 'min'**)**  max2 **<-** cellStats**(**r, 'max'**)**  r **<-** raster**(**fls**[**3**])**  min3 **<-** cellStats**(**r, 'min'**)**  max3 **<-** cellStats**(**r, 'max'**)**  r **<-** raster**(**fls**[**4**])**  min4 **<-** cellStats**(**r, 'min'**)**  max4 **<-** cellStats**(**r, 'max'**)**  r **<-** raster**(**fls**[**5**])**  min5 **<-** cellStats**(**r, 'min'**)**  max5 **<-** cellStats**(**r, 'max'**)**  r **<-** raster**(**fls**[**6**])**  min6 **<-** cellStats**(**r, 'min'**)**  max6 **<-** cellStats**(**r, 'max'**)**  minval **<-** rbind**(**min1, min2, min3, min4, min5, min6**)**  maxval **<-** rbind**(**max1, max2, max3, max4, max5, max6**)**  mm.df **<-** data.frame**(**bricknames, minval, maxval**)**  #binary training raster  Owetland **<-** raster**(**"J:/LandCover/OrganicWetlandProbability/Training/Owetland.tif"**)**  #----------------------------------------------------------------  #----------------------------------------------------------------  #################################################################  #BUILD MODEL 1  ###################################################################  #------------------------------------------------------------------  #------------------------------------------------------------------  #define fit AUC and dev  AUC **<-** vector**()**  dev **<-** vector**()**  setwd**(**location.pts**)**  dat **<-** read.csv**(**"d1.csv"**)**  dat **<-** dat**[**,**-**c**(**4,8**)]**  #defint model list  fit **<-** list**()**  #build model  fit**[[**1**]]** **<-** gbm.step**(**dat, 1**:**length**(**fls**)**, length**(**fls**)+**1, family **=** "bernoulli", tree.complexity **=** 8,  learning.rate **=** 0.005, bag.fraction **=** 0.5, silent **=** **TRUE**, warnings **=** **FALSE)**  df.importance **<-** data.frame**(**summary**(**fit**[[**1**]]))**  df.importance **<-** arrange**(**df.importance, var**)**  df.importance **<-** df.importance**[**,2**]**  #model stats  AUC**[**1**]** **<-** fit**[[**1**]]$**cv.statistics**$**discrimination.mean  dev**[**1**]** **<-** **(**fit**[[**1**]]$**self.statistics**$**mean.null **-** fit**[[**1**]]$**self.statistics**$**mean.resid**)** **/** fit**[[**1**]]$**self.statistics**$**mean.null    response.df **<-** data.frame**(**dummy**=**c**(**1**:**1001**))**  **for** **(**n **in** bricknames**){**  d **<-** plot.gbm**(**fit**[[**1**]]**, i.var **=** n, return.grid**=TRUE**, type**=**"response"**)**  get.min.max **<-** filter**(**mm.df, bricknames **==** n**)**  mn **<-** get.min.max**[**,2**]**  mx **<-** get.min.max**[**,3**]**  xout **<-** seq**(**mn, mx, **(**mx**-**mn**)/**1000**)**  d **<-** approx**(**d**[**,1**]**, d**[**,2**]**, xout **=** xout, rule**=**2**)**  d **<-** as.data.frame**(**d**)**  response.df **<-** cbind**(**response.df,d**)**  **}**  #----------------------------------------------------------------  #----------------------------------------------------------------  #################################################################  #BUILD MODEL ALL MODELS AND OUTPUT STATS  ###################################################################  #------------------------------------------------------------------  #------------------------------------------------------------------  setwd**(**location**)**  **for** **(**i **in** 2**:**iter**){**  setwd**(**location.pts**)**  dat **<-** read.csv**(**paste0**(**"d", i, ".csv"**))**  dat **<-** dat**[**,**-**c**(**4,8**)]**    #build model  fit**[[**i**]]** **<-** gbm.step**(**dat, 1**:**length**(**fls**)**, length**(**fls**)+**1, family **=** "bernoulli", tree.complexity **=** 8,  learning.rate **=** 0.005, bag.fraction **=** 0.5, silent **=** **TRUE**, warnings **=** **FALSE)**  v.importance **<-** as.data.frame**(**summary**(**fit**[[**i**]]))**  v.importance **<-** arrange**(**v.importance, var**)**  v.importance **<-** v.importance**[**,2**]**  df.importance **<-** cbind**(**df.importance, v.importance**)**    **for** **(**n **in** bricknames**){**  d **<-** plot.gbm**(**fit**[[**i**]]**, i.var **=** n, return.grid**=TRUE**, type**=**"response"**)**  get.min.max **<-** filter**(**mm.df, bricknames **==** n**)**  mn **<-** get.min.max**[**,2**]**  mx **<-** get.min.max**[**,3**]**  xout **<-** seq**(**mn, mx, **(**mx**-**mn**)/**1000**)**  d **<-** approx**(**d**[**,1**]**, d**[**,2**]**, xout **=** xout, rule**=**2**)**  d **<-** as.data.frame**(**d**)**  response.df **<-** cbind**(**response.df,d**)**  **}**    #model stats  AUC**[**i**]** **<-** fit**[[**i**]]$**cv.statistics**$**discrimination.mean  dev**[**i**]** **<-** **(**fit**[[**i**]]$**self.statistics**$**mean.null **-** fit**[[**i**]]$**self.statistics**$**mean.resid**)** **/** fit**[[**i**]]$**self.statistics**$**mean.null    print**(**paste0**(**"done building model ", i**))**  **}**  importance **<-** rowMeans**(**df.importance**)**  imp.names **<-** c**(**"ARI", "NDPOL", "PC1", "REIP", "TPI", "TWI"**)**  imp.df **<-** cbind**(**as.numeric**(**df.importance**)**, imp.names**)**  importance **<-** data.frame**(**imp.names, importance**)**  #----------------------------------------------------------------  #----------------------------------------------------------------  #################################################################  #GENERATE RESPONSE CURVES AND MODEL STATS  ###################################################################  #------------------------------------------------------------------  #------------------------------------------------------------------  response.df **<-** response.df**[**,**-**1**]**  response.df.x **<-** response.df**[**,seq**(**1,length**(**response.df**)**,2**)]**  response.df.x **<-** response.df.x**[**,1**:**length**(**bricknames**)]**  response.df.y **<-** response.df**[**,seq**(**2,length**(**response.df**)**,2**)]**  **for** **(**i **in** 1**:**length**(**bricknames**)){**  varname **<-** bricknames**[**i**]**  collumns **<-** seq**(**i, length**(**response.df.y**)**, length**(**bricknames**))**  yvals **<-** response.df.y**[**,collumns**]**  xvals **<-** response.df.x**[**,i**]**  yvals.mean **<-** rowMeans**(**yvals**)**  yvals.std **<-** apply**(**yvals,1,sd**)**  yvals.neg.std **<-** yvals.mean **-** yvals.std  yvals.add.std **<-** yvals.mean **+** yvals.std  yvals.df **<-** cbind**(**xvals, yvals.mean, yvals.neg.std, yvals.add.std**)**  yvals.df **<-** as.data.frame**(**yvals.df**)**  **if(**i**>**0**){**  xlim **<-** c**(**min**(**xvals**)**, max**(**xvals**))**  **}** **else{**  xlim **<-** c**(**min**(**xvals**)**, 1200**)**  **}**  g **<-** ggplot**(**yvals.df, aes**(**x**=**xvals, y**=**yvals.mean**))** **+**  theme_minimal**()+**  geom_ribbon**(**aes**(**ymin**=**yvals.neg.std, ymax**=**yvals.add.std**)**, fill**=**"#6baed6", alpha**=**0.35**)** **+**  geom_line**(**colour**=**"#08519c", size**=**1.8**)** **+**  xlab**(**varname**)** **+** ylab**(**"predicted probability"**)** **+** xlim**(**xlim**)** **+**  theme**(**axis.title.x **=** element_text**(**size**=**22**)**, axis.title.y **=** element_text**(**size**=**20**)**,axis.text **=** element_text**(**size**=**16**))**  assign**(**paste0**(**varname,".plot"**)**, g**)**  **}**  #output model stats  setwd**(**outputs**)**  AUC **<-** mean**(**AUC**)**  dev **<-** mean**(**dev**)**  stats **<-** cbind**(**AUC,dev**)**  write.csv**(**stats, paste0**(**"OwetlandModelStats.csv"**)**, row.names**=FALSE)**  tiff**(**paste0**(**"OwetlandResponseCurves.tiff"**)**, width **=** 1200, height **=** 1000**)**  multiplot**(**ARI.plot, NDPOL.plot, PC1.plot, REIP.plot, TPI.plot, TWI.plot, cols**=**2**)**  dev.off**()**  tiff**(**paste0**(**"OwetlandVarImportance.tiff"**)**, width **=** 1000, height **=** 700**)**  ggplot**(**importance,aes**(**x**=**reorder**(**imp.names,**-**importance**)**,y**=**importance**))** **+** geom_bar**(**stat**=**"identity", show.legend**=FALSE**, fill**=** "grey60"**)** **+** theme_minimal**()** **+**  theme**(**axis.title.x **=** element_text**(**size**=**24**)**, axis.title.y **=** element_text**(**size**=**24**)**,axis.text **=** element_text**(**size**=**22**)**, legend.text **=** element_text**(**size**=**20**)**, legend.title **=** element_text**(**size**=**22**))** **+**  labs**(**x **=** "Variables"**)** **+** labs**(**y **=** "Importance"**)**  dev.off**()**  #----------------------------------------------------------------  #----------------------------------------------------------------  #################################################################  ###################################################################  #SECTION 2 PREDICT WETLAND PROBABILITY BY TILE  ###################################################################  #LOOP THROUGH PREDICTION OF RASTERS BASED ON MODEL FITS  ###################################################################  #------------------------------------------------------------------  #------------------------------------------------------------------  PUs **<-** readOGR**(**"J:/LandCover/CurrentSurfaceWater/Boreal", "Boreal_PUs"**)**  **for** **(**i **in** 1**:**length**(**PUs**)){**  t1 **<-** Sys.time**()**    #build raster brick  setwd**(**location**)**  fls **<-** tifs  PU **<-** PUs**[**i,**]**  #build raster brick  r **<-** raster**(**fls**[**1**])**  r1 **<-** crop**(**r, PU**)**  r **<-** raster**(**fls**[**2**])**  r2 **<-** crop**(**r, PU**)**  r **<-** raster**(**fls**[**3**])**  r3 **<-** crop**(**r, PU**)**  r **<-** raster**(**fls**[**4**])**  r4 **<-** crop**(**r, PU**)**    r **<-** raster**(**fls**[**5**])**  r5 **<-** crop**(**r, PU**)**    r **<-** raster**(**fls**[**6**])**  r6 **<-** crop**(**r, PU**)**    r.b **<-** brick**(**r1,r2,r3,r4, r5, r6**)**  names**(**r.b**)** **<-** bricknames    #predict fit[[1]]  beginCluster**(**9**)**  r.b.p **<-** clusterR**(**r.b, raster**::**predict, args **=** list**(**model **=** fit**[[**1**]]**, type **=** "response", n.trees **=** fit**[[**1**]]$**gbm.call**$**best.trees**))**  endCluster**()**  plot**(**r.b.p**)**    #START PREDICTION OF RASTER STACK  **for** **(**n **in** 2**:**length**(**fit**)){**  beginCluster**(**9**)**  prediction **<-** clusterR**(**r.b, raster**::**predict, args **=** list**(**model **=** fit**[[**n**]]**, type **=** "response", n.trees **=** fit**[[**n**]]$**gbm.call**$**best.trees**))**  endCluster**()**  r.b.p **<-** stack**(**r.b.p, prediction**)**  print**(**paste0**(**"Done model prediction ", n**))**  **}**    OwetlandProbability **<-** calc**(**r.b.p, fun **=** mean**)**  OwetlandProbability.sd **<-** calc**(**r.b.p, fun **=** sd**)**    #Save wetland prediction rasters and standar deviation between the 40 models  setwd**(**outputs**)**  writeRaster**(**OwetlandProbability, paste0**(**"OwetlandProbability", i, ".tif"**)**, datatype**=** "FLT4S"**)**  writeRaster**(**OwetlandProbability.sd, paste0**(**"OwetlandProbabilitySD", i, ".tif"**)**, datatype**=** "FLT4S"**)**    t2 **<-** Sys.time**()**  t.diff **<-** difftime**(**t2,t1, units**=**"hours"**)**  print**(**paste0**(**"Done predicting PU ", i, " it took ", round**(**t.diff, 2**)**, " hours"**))**    **}**  #------------------------------------------------------------------  #------------------------------------------------------------------  ####################################################################  tt2 **<-** Sys.time**()**  t.diff **<-** difftime**(**tt2,tt1, units**=**"hours"**)**  print**(**paste0**(**"total script took ", round**(**t.diff, 2**)**, " hours"**))** |
| --- |
