# Supplementary figures and images for: Large-scale probabilistic identification of boreal peatlands using Google Earth Engine, open-access satellite data, and machine learning
Source: PLoS One. 2019 Jun 17;14(6):e0218165. doi: 10.1371/journal.pone.0218165 (PMC6576777; doi:10.1371/journal.pone.0218165)

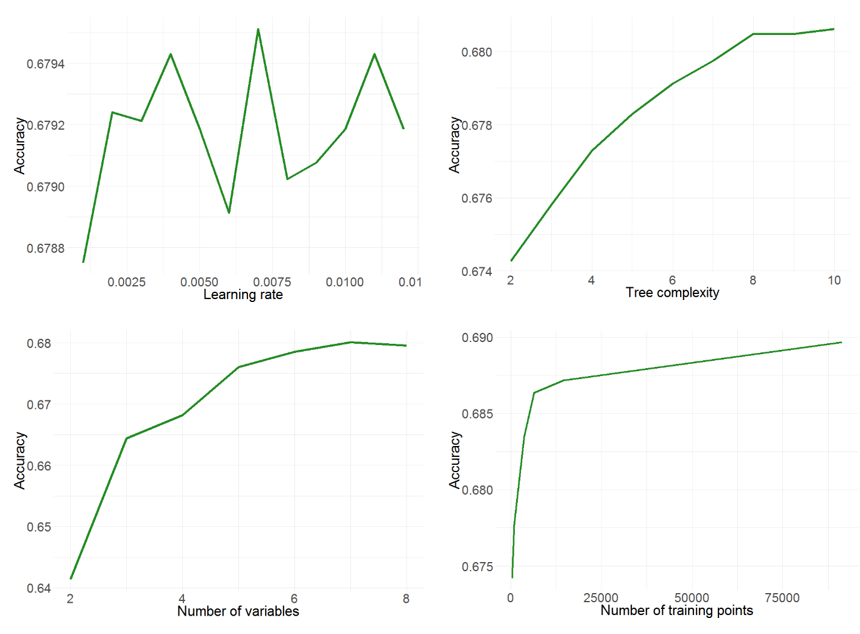

Supplement: S1 Data — The ABMI has given permission to publish this image under a CC BY 4.0 license. (TIFF) [file pone.0218165.s002.tiff]
